# The Tumor-on-Chip: Recent Advances in the Development of Microfluidic Systems to Recapitulate the Physiology of Solid Tumors

**DOI:** 10.3390/ma12182945

**Published:** 2019-09-11

**Authors:** Grissel Trujillo-de Santiago, Brenda Giselle Flores-Garza, Jorge Alfonso Tavares-Negrete, Itzel Montserrat Lara-Mayorga, Ivonne González-Gamboa, Yu Shrike Zhang, Augusto Rojas-Martínez, Rocío Ortiz-López, Mario Moisés Álvarez

**Affiliations:** 1 Centro de Biotecnología-FEMSA, Tecnologico de Monterrey, Monterrey, Nuevo León CP 64849, Mexico; 2 Departamento de Ingeniería Mecátrónica y Eléctrica, Tecnologico de Monterrey, Monterrey, Nuevo León CP 64849, Mexico; 3 Division of Engineering in Medicine, Department of Medicine, Brigham and Women’s Hospital, Harvard Medical School, Cambridge, MA 02139, USA; 4 Centro de Investigación y Transferencia en Salud, Hospital San José, Tecnologico de Monterrey, Monterrey, Nuevo León CP 64849, Mexico

**Keywords:** tumor-on-a-chip, cancer, organ-on-a-chip, microfluidics, nanoparticles, tissue engineering

## Abstract

The ideal in vitro recreation of the micro-tumor niche—although much needed for a better understanding of cancer etiology and development of better anticancer therapies—is highly challenging. Tumors are complex three-dimensional (3D) tissues that establish a dynamic cross-talk with the surrounding tissues through complex chemical signaling. An extensive body of experimental evidence has established that 3D culture systems more closely recapitulate the architecture and the physiology of human solid tumors when compared with traditional 2D systems. Moreover, conventional 3D culture systems fail to recreate the dynamics of the tumor niche. Tumor-on-chip systems, which are microfluidic devices that aim to recreate relevant features of the tumor physiology, have recently emerged as powerful tools in cancer research. In tumor-on-chip systems, the use of microfluidics adds another dimension of physiological mimicry by allowing a continuous feed of nutrients (and pharmaceutical compounds). Here, we discuss recently published literature related to the culture of solid tumor-like tissues in microfluidic systems (tumor-on-chip devices). Our aim is to provide the readers with an overview of the state of the art on this particular theme and to illustrate the toolbox available today for engineering tumor-like structures (and their environments) in microfluidic devices. The suitability of tumor-on-chip devices is increasing in many areas of cancer research, including the study of the physiology of solid tumors, the screening of novel anticancer pharmaceutical compounds before resourcing to animal models, and the development of personalized treatments. In the years to come, additive manufacturing (3D bioprinting and 3D printing), computational fluid dynamics, and medium- to high-throughput omics will become powerful enablers of a new wave of more sophisticated and effective tumor-on-chip devices.

## 1. Introduction

Cancer continues to be one of the most important causes of mortality across the globe [1,2,3]. Estimates from the World Health Organization (WHO) indicate that cancer is either the first or the second cause of mortality in 91 of 172 countries before 70 years of age. In an additional set of more than 20 countries, cancer ranks third or fourth as a cause of death [1]. Yearly, more than 18 million patients will be diagnosed with cancer, and approximately 9.5 million will die from this disease, according to GLOBOCAN 2018 estimates [1]. Cancer trends are also worrisome, and the number of patients diagnosed with cancer continues to grow. In this century, cancer will most probably rank as the single most important hurdle to increasing life expectancy and the main cause of death in every region of the world.

**Figure 1:**
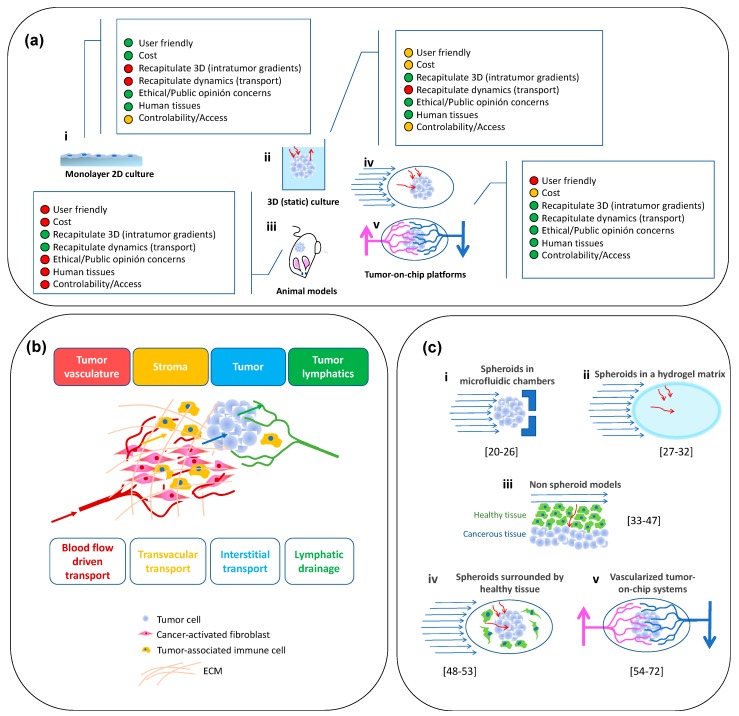
Recapitulation of the tumor niche using tumor-on-chip systems with various degrees of sophistication. (**a**) The evolution of our cancer research tools—from 2D to 3D, and beyond—tumor-on-chip systems. Colored circles show the degree of fulfillment of different features. Green: fully fulfilled; Yellow: partially fulfilled; Red: poorly fulfilled; (**b**) Scheme of the tumor niche. Modified from Han et al., 2016 [19]. (**c**) Different types of tumor-on-chip systems: from simple spheroids in a microflow to vascularized and perfusable tumor microtissues [20,21,22,23,24,25,26,27,28,29,30,31,32,33,34,35,36,37,38,39,40,41,42,43,44,45,46,47,48,49,50,51,52,53,54,55,56,57,58,59,60,61,62,63,64,65,66,67,68,69,70,71,72].

These numbers impose great pressure on research groups and the pharmaceutical industry to identify more and better drugs that are effective against cancer [4,5,6]. Today, less than 10% of the anticancer drugs that enter clinical trials ever reach the market [7,8]. Not infrequently, anticancer pharmaceuticals fail during clinical development, even after showing good potential in extensive preclinical testing. This strongly suggests that the current in vitro and preclinical models are not reliable predictors of the actual in vivo efficacy and toxicity of anticancer drugs in humans [8] (Figure 1a). Therefore, we urgently require reliable models that are capable of a more precise recapitulation of the effects of anticancer therapeutics in humans and that will increase the success rate of movement of candidate drugs through the pipeline of anticancer drug development.

Recently, organ-on-chip systems—microphysiological systems that combine the use of microfluidics, tissue engineering, and microfabrication tools—have shown promise sustaining functional microtissues for relatively extended timeframes [9,10,11,12]. Tumor-on-chip models based on these organ-on-chip systems can recreate human tumor microenvironments and now hold great promise as a new resource for cost-effective and higher-throughput screening of anticancer drugs [13,14,15,16,17] and powerful enablers of precision medicine [18].

In this review, we will focus on solid tumors and their recapitulation in tumor-on-chip systems. Our discussions revolve mainly around the relevant data published in the last 8 years (from 2011 to date) related to the culture of tumor-like structures (not isolated cancer cells) in microfluidic systems. We primarily adopt an engineering/fabrication angle, and we emphasize the materials, architecture, and geometric and operational features that enable cancer research in these microfluidic devices. In that sense, our intent is to provide a tutorial review that introduces a wide portfolio of ideas for the engineering of cancer-on-chip systems with various degrees of sophistication and with a focus on the recapitulation of solid tumors.

We set the stage by describing the tumor niche (Figure 1b; Section 2) and by discussing (in more detail) the need for, and advantages of, recreating the tumor architecture and physiology in tumor-on-chip platforms (Section 3). Then, we follow a rationale of increasing architecture complexity (Figure 1c). We start by discussing simple geometrical arrays: devices in which cancerous microtissues are cultured in some sort of 3D architecture. We then move up in scale to papers that discuss the interaction of 3D cancerous and healthy tissues. Subsequently, we review a series of contributions describing the use of tumor spheroids with an architecture resembling that of actual tumors to recreate, for example, the transport of nanoparticles and drugs to the interior of a tumorous tissue. We then discuss the tumor-on-chip systems in which tumor microtissues (not necessarily in the form of spheroids) are surrounded by healthy cells and/or tissues. Finally, we discuss even more realistic systems where the cancerous tissue is surrounded by both an extracellular matrix and healthy cells and contains vasculature that perfuses nutrients through it.

## 2. The Tumor Niche

In general, tumor tissues are composed of multiple cell types (i.e., cancer cells, various stromal cells, such as cancer-associated fibroblasts [73], various types of immune cells, and vascular cells) and rich extracellular matrix components (i.e., type I collagen) [25,74] (Figure 1c). In addition to their remarkable internal heterogeneity, the tumor stromal tissues (i.e., the tissues surrounding the tumor), act as a dynamic source (and reservoir) of various cytokines and growth factors that influence tumor progression and pharmacological responses [19]. Cancer–stroma interactions have effects in many aspects of tumor behavior, including tumorigenesis, angiogenesis, tumor invasion, metastasis, and resistance to therapeutic agents [73,74]. Many molecules produced by cancerous or stromal cells dictate the growth dynamics of solid tumors. For example, the role of E-cadherin as a suppressor of cancer invasiveness has been widely cited [75,76]. Other cellular components—for example, immune cells—play key roles in the evolution of a tumor (i.e., the presence of M2 macrophages accelerates tumor progression) [77,78,79,80]. Despite almost two decades of accelerating research progress, the complex proteomics and metabolomics of cancer are yet to be understood in detail. 

Another evident and relevant particularity of solid tumors is their 3D nature. Three-dimensionality has been confirmed to have a paramount importance in the proper understanding of tumor dynamics. For instance, recently published literature discusses the role of the size of a tumor (and the associated hypoxia levels) on its aggressiveness [81,82,83,84,85] and on the efficacy of drug delivery to it [84,86,87].

Overall, cancer is truly a set of complex pathologies that share some common features (e.g., uncontrolled emergence of mutations and growth of malignant cells). However, each cancer type exhibits particularities that greatly complicate the development and selection of adequate therapies for that unique cancer type. In the end, cancer is a genetic disorder [88,89]. Cancer cells acquire and accumulate mutations in an uncontrolled manner. The nature of these mutations differs for each cancer [90] and in each individual [90,91,92]. At the extreme, each cell in a particular tumor may exhibit a different set of mutations [91].

## 3. Tumors-On-Chips: A Superior Alternative for Emulating the Tumor Micro-Niche

Several convincing arguments favor the use of tumor-on-chip models as tools for solid-tumor research. In brief, these arguments are related to the following three relevant characteristics of solid tumors: (a) their 3D nature, (b) their structural and dynamical complexity, and (c) the fact that they are not easily accessible/observable (in animal models and human patients).

The conventional experimental platforms widely available today to conduct cancer research exhibit limitations related to these three aspects. Figure 1a presents the evolution of solid-tumor research tools, from two-dimensional (2D) monolayer cell cultures to 3D culture systems and animal models to tumor-on-chip systems. In a graphical way, we describe the main attributes of each of these research platforms. While 2D systems were the first workhorses in cancer research, a vast body of experimental evidence confirms that the relevant features of the cancer progression, and the effectiveness of anticancer pharmaceutical compounds, cannot be properly recapitulated in 2D cell culture systems [8,57,93]. By contrast, animal models are research platforms that satisfy the 3D requirement [94,95] and provide an in vivo setting to study tumor growth, behavior, and treatment. However, animal models have their own complications [7,17,96] because they are time-, resource-, and expertise-intensive, and animal use is increasingly being questioned by different public entities for ethical/humanitarian concerns [97,98,99]. Besides, animal models typically fail to provide a precise recapitulation of human physiology [74,100,101].

The conventional in vitro 3D cancer models are static systems (i.e., they are not based on microfluidics) [102], but they do provide a valuable alternative to animal models and have enabled important advances in cancer research in the last two decades [5,103]. Nevertheless, the widespread use of 3D cell cultures into cancer research is still limited by various factors, including their reproducibility and cost [5,17]. Current 3D culture methodologies do not fully capture the complexity of the in vivo environment, simply because they do not consider the dynamic component. Today, the authentic replication of the dynamic environment is recognized as a key element in the proper evaluation of the efficiency and specificity of anticancer drugs.

Microfluidics offers the possibility of maintaining and studying primary or microfabricated tissue samples in a controlled environment while recreating (at least to some extent) the physiological dynamic characteristics [104]. Many aspects of the biology of cancer have been studied in microfluidic systems in which cancerous cells (frequently co-cultured with non-cancerous cells) are cultured under well-controlled conditions [8,105,106,107,108]. Recent reviews have described the current use of microfluidics to investigate different aspects of the physiology of cancer, including (a) cell migration in the tumor microenvironment [105,109], metastasis events [110], anticancer drug screening and therapy response predictions [111], or the study of the transport of anticancer nanomedicines in tumorous tissues [112].

As previously stated, in this review, we focus on the recent development of in vitro microfluidic solid-tumor platforms, or tumor-on-chip systems [8,88,113]. These tumor-on-chip systems are expected to increase the effective identification of new and better cancer therapies while minimizing non-specific toxicity [13,14,15,16,17]. Tumor-on-chip systems are also finding relevant niches of application as fundamental cancer research tools [104]. In the very near future, tumor-on-chip systems promise to be powerful enablers of anticancer personalized/precision medicine [18]. Today, tumor-on-chip systems are the most promising embodiment of in vitro platforms capable of faithfully recapitulation of relevant aspects of the biochemical complexity and dynamics of the tumor niche in a controlled environment (i.e., with much lower variability than is associated with in vivo systems [8,54,113]).

## 4. Tumor Spheroids: Fabrication Techniques

The fabrication of spheroids is often a prerequisite for the development of tumor-on-chip platforms. Cancer spheroids are arguably the simplest in vitro 3D tumor models [114,115]. Despite their simplicity, they hold great promise for the recapitulation of solid tumors in many relevant aspects [22,29,116,117]. Unlike monolayer cell cultures, these single-cell type or multi-cell type cellular bodies [118,119] resemble the 3D architecture of actual solid tumors, show an organized cellular architecture, and exhibit realistic cell-cell and cell-extra cellular matrix (ECM) interactions [115,120] as compared to 2D culture systems.

**Figure 2:**
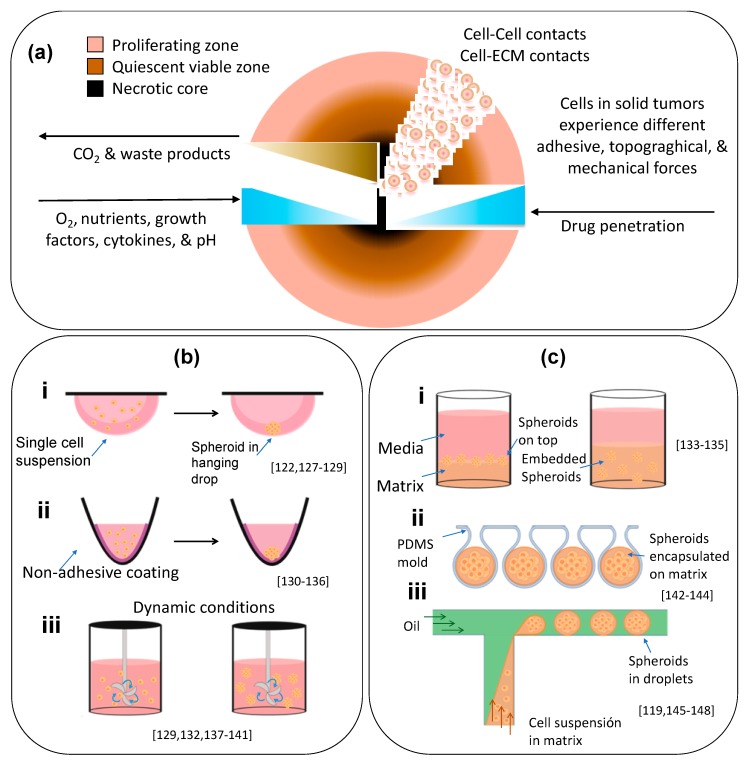
Architecture of tumor spheroids and their fabrication techniques. (**a**) Schematic representation of the 3D architecture of a tumor spheroid. Modified from Sant and Johnston 2017 [126]; (**b**) Fabrication of spheroids by the following scaffold-free methods: (i) hanging drop [122,127,128,129], (ii) liquid-overlay method [130,131,132,133,134,135,136], and (iii) force-driven method [129,132,137,138,139,140,141]; (**c**) Fabrication of spheroids by the following scaffold-based methods: (i) spheroids with a matrix-on top and embedded in a matrix [133,134,135], (ii) spheroids encapsulated within a molded matrix [142,143,144], and (iii) spheroids formed by suspending cancer cells within a liquid matrix and applying microfluidic-based strategies to form spheroids in droplets [119,145,146,147,148].

Moreover, in close resemblance to non-vascularized or poorly vascularized tumors, spheroids exhibit intrinsic metabolic (nutrients, oxygen, carbon dioxide, and byproduct) gradients that lead to the establishment of a multi-layered structure (i.e., an external layer comprised by proliferative cells, an intermediate layer composed of mainly quiescent cells, and an inner layer, hypoxic and acidic, mainly constituted by necrotic cells) [115,116] (Figure 2a). Several studies have shown that tumor spheroids display an enhanced deposition of tumor ECM proteins (e.g., fibronectin, lumican, laminin, and collagen type I and VI) [118,121,122,123] in comparison to 2D cell culture models. Spheroids can also be fabricated to contain different cell types, as actually occurs in the tumor niche, so that a realistic cell–cell chemical signaling and cell–cell physical interactions may be established. In sum, spheroids closely mimic some of the relevant characteristics of human solid tumors, including the presence of a nutrient and oxygen gradients, a multi-cellular composition, and their layered architecture. These features confer to spheroids a similar anticancer drug resistance profile [124,125] to that exhibited by real solid tumors.

Several approaches can be used for the in vitro fabrication of spheroids ranging from conventional methods using dispersed cells to more sophisticated microfluidic platforms (Figure 2b) [126]. Spheroids can form with or without a matrix support (i.e., scaffold-based or scaffold-free fabrication) [124]. Scaffold-based methods support the 3D organization of the cells and are commonly used for tissue engineering applications. Scaffold-free methods for spheroid production are most commonly used because they are relatively inexpensive, rapid, and user-friendly.

In general, these spheroids form because the methods used promote the aggregation of the cells and direct cell-cell contact. Under very simple culture conditions, tumor cells spontaneously aggregate to form spheroids. This formation takes place in three stages [127]: disperse cells first form loose aggregates through the binding of their ECM fibers (highly loaded with RGD motifs) that act as long-chain linkers of cell membrane integrins; second, direct cell-cell contact upregulates the expression of E-cadherin, which accumulates in the cell surface; and third, the spheroid becomes compacted due to the strong homophilic interactions between the E-cadherin molecules of the cells.

Scaffold-free methods include the liquid-overlay technique, the hanging drop method, and agitation-based and other force-driven methods. In general, these methods are widely used due to their simplicity and cost-effectiveness. In hanging drop protocols, spheroids form by gravity, as cancer cells aggregate into small spheroids within hanging liquid drops of relatively dense cell suspensions that are incubated upside-down [149]. Hanging drop methods have limited working volumes of 20–50 μL. Consequently, they require frequent medium replacement to prevent dehydration, which makes these methods labor-intensive, unstable, and unlikely to produce large spheroids. To overcome these inherent difficulties, Lee et al. developed a long-term hanging drop method that used a soft lithographic approach [128]. They created hollow spheres by injecting liquid drops into non-cured polydimethylsiloxane (PDMS) mixtures. This cell culture method supported larger media volumes of up to 500 μL, enabling the growth of spheroids without significant media depletion over 10 to 15 days. Clever integrations of the hanging drop method and a microfluidic manifold for medium- to high-throughput spheroid generation have also been recently proposed [150,151].

Other liquid-overlay culture methods rely on preventing adhesion of the cells to culture surfaces to favor cell-cell adhesion and the formation of aggregates [115,152]. Adhesion can be prevented by coating the culture surface with agar [153], agarose [120,130,152,154], poly (2-hydroxyethyl methacrylate) (polyHEMA) [125], or tri-block co-polymers, such as Pluronic (F108) [26]. Other materials have been confirmed as suitable non-adherent surfaces due to their topography [155] or high hydrophilicity. For example, the commercially available Ultra Low Attachment (ULA) microwell culture plates have become a popular resource for fabricating spheroids [20,126,156], as has hydrophilic filter paper, which enables the spontaneous formation of prostate cancer spheroids and a significant enrichment of the cancer stem cell (CSC) population using regular cell culture medium [131].

Tumor spheroids can also be formed under dynamic conditions. Benign agitation or rotational flows hinder cell-substrate contact, thereby promoting the formation of cell aggregates. Spinner and rotational flasks have been commonly used [137,138] to fabricate spheroids under benign agitation. The spinner system contains a magnetic stirrer that prevents cell adhesion while distributing nutrients and O_2_ throughout the culture medium. By contrast, rotational culture uses a slow rotation of the culture flask to create a microgravity environment that keeps cells in suspension while allowing them to aggregate into spheroids. These methods are useful for the large-scale production of spheroids. However, they require large amounts of culture medium, they do not allow control of the size of the generated spheroids, and the shear force of the agitation can be harmful to the cells [114,115,157].

Other scaffold-free methods have been recently reported—for example, the force-driven formation of spheroids by dielectrophoresis using interdigitated microelectrodes [139], the use of acoustic bulk and surface waves [140,141], and the magnetically assisted fabrication of spheroids labeled with paramagnetic particles [129,158], which enables cell immobilization using magnetic fields and simplifies handling and high-throughput screening during drug testing [132].

Spheroids can be fabricated using scaffold-based methods by three main strategies: matrix-on-top, matrix-embedded, and matrix encapsulation methods. In the first two, the cells are seeded on top of the matrix or within the matrix upon gelation, respectively, whereas matrix encapsulation protocols employ microfluidic platforms. Matrix-based methods use natural or synthetic hydrogels that can be adjusted to resemble the tumor microenvironment. The biophysical and biochemical properties of hydrogels typically used to fabricate cancer spheroids have been widely studied and are discussed elsewhere [159,160,161]. Briefly, natural polymers are similar to the ECM of real tumors; they are rich in nutrients, biocompatible, biodegradable, and allow cell signal transduction. However, they have poor mechanical and limited tunable properties, they tend to degrade rapidly, and they have low reproducibility due to batch-to-batch variations. Nevertheless, recent papers illustrate the use of naturally derived hydrogels for spheroid fabrication, such as collagen [122,162], Matrigel [142,143], alginate [146], and hyaluronic acid [163], and combinations of these and other materials [134,164]. Synthetic polymers have design flexibility, tunable mechanical properties, and assay reproducibility. The most commonly used synthetic polymer is poly (ethylene glycol) (PEG) and its derivatives [114,165]. Several techniques also involve the use of semi-synthetic polymers, such as gelatin methacryloyl (GelMA) [166] based hydrogels [135,167], or self-assembling peptides, such as RADA16-I, Q11, and bQ13, that can form gels under specific conditions (e.g., pH) [136]. The opacity of some gels, their different diffusion rates for nutrients and compounds, the dispersed Z-plane location of the spheroids, and the control of the shape and size of spheroids, present challenges for the analysis and capture of data and for the use of high-throughput screening of drugs in scaffold-based methods.

The population of spheroids produced by 3D cell culture techniques is often fairly disperse in terms of size and shape, which can strongly influence the outcome of drug efficacy and toxicity studies. The development of standardized spheroid fabrication procedures could potentially reduce data variability and enhance the clinical significance of experimental data derived from spheroid systems [5]. Recently, several bioprinting and microfluidic-based strategies have been developed that aim to minimize the variations in spheroid size and shape. A few bioprinting techniques have been reported for the fabrication of tumor spheroids [93,142,144,168]. Recent reports have described the high-throughput and continuous fabrication of tumor spheroids of tunable size by microfluidic means [57,119,169,170].

Although other methods have been proposed [150,151,171,172], the generation of spheroids by microfluidic techniques is done mainly by approaches based on micro-molding and micro-droplets. The micro-molding method uses lithography to fabricate complex structures, made of natural or synthetic polymers, to form spheroids inside the microfluidic device. The end result is a reduction in the consumption of reagents, a higher density of cells, a higher cell to fluid ratio, and real-time control of delivery of nutrients and reagents [20,124,145,173,174]. By contrast, the micro-droplet generation technique uses the microfluidic device to produce homogenously sized droplets of hydrogel-embedded cells in a continuous flow of an oleaginous solution. The hydrogel droplets can be gelled by temperature, light, or ion treatments which allows a large-scale production of uniformly sized, encapsulated spheroids with minimal user-device interaction [147,169,175].

Microfluidic approaches [176] hold several advantages over conventional methods for spheroid fabrication, such as higher throughput, continuous production, precise control of the pressure gradients and sheer stresses to which cells will be exposed, less reagent consumption, and uniformity of spheroid shape and size. Overall, these characteristics make microfluidics-based methodologies highly attractive for the fabrication of homogeneous populations of spheroids for cancer research.

## 5. Tumor-on-Chip Examples

### 5.1. Spheroids in Microfluidic Chambers

Arguably, the simplest tumor-on-chip system would consist of a tumor spheroid directly placed under flow in a microfluidic system [20,21,22,23,24,25,26]. Although this might seem to be a radical oversimplification of the tumor niche, this straightforward system actually recapitulates the 3D nature of real tumors and may be appropriate for gaining some insight into the biology of human tumors for specific application scenarios.

**Figure 3:**
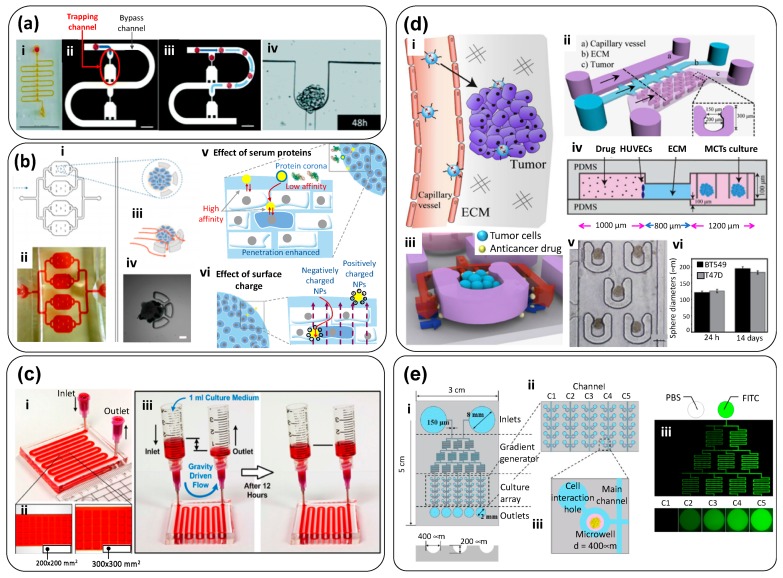
Simple tumor-on-chip systems: a tumor spheroid continuously perfused within a microfluidic device. (**a**) Simple serpentine-shaped microfluidic system designed by Ruppen et al. [20], (i) designed to trap cancer cell agglomerates for fabrication of tumor spheroids. A simple hydraulic system enabled the trapping of cell agglomerates (scale bar: 10 μm) (ii) in small cavities. Once the upstream cavities were occupied by spheroids, flow was directed to downstream portions of the serpentine (scale bar: 500 μm) (iii) to favor new trapping events. (iv) Spheroids grew and matured in these cavities and were continuously exposed to anticancer compounds. Taken from reference [20]; (**b**) Microfluidic device developed by Huang et al. [21]: (i) general architecture, (ii) hydraulic testing, (iii) detail of the spheroid traps, and (iv) actual spheroid trapped in a holder as imaged in bright field. Schematic representation of the effect of (scale bar: 50 μm) (v) protein corona, and (vi) charge, on nanoparticle penetration into tumor spheroids. Taken from reference [21]; (**c**) Simple pump-independent (i) microfluidic system devised by Patra et al. [22] for continuous formation and perfusion of cancer spheroids (ii) contained within rectangular cavities located at the floor of the microfluidic circuit. (iii) Flow is driven simply by a pressure head induced by a difference in the height of the column of liquid (culture medium) between the inlet and the outlet reservoir. Taken from reference [22]; (**d**) Tumor-on-chip system developed by Chen et al. [177]: (i) Scheme of the physiological context aimed, (ii) and overall microfluidic architecture. (iii) 3D-render showing cancer cells retained within a horseshoe trap. Red and blue arrows indicate the flow fields within the system. (iv) Scheme of the architecture of the system (side view), (v) detail of the array of traps holding spheroids of consistent size, and (vi) size of spheroids after 14 days of culture. Scale bar: 200 μm. Taken from reference [177]; (**e**) The microfluidic system developed by Lim and Park [23] enables the exposure of cancer spheroids to anticancer drug gradients. (i) Scheme of the overall architecture of the device, (ii) detail of the cell-culture section, and (iii) close-up showing the geometry of each culture microwell. (iv) Experimental demonstration of the effective generation of gradients using injections of a fluorescent tracer. Taken from reference [23].

One of the earliest examples of a tumor-on-chip system was presented by Ruppen et al. [20]. These authors trapped cancer spheroids derived from malignant pleural mesothelioma (MPM) cells within a “multi-S-shaped” microfluidic channel (Figure 3a) and perfused culture medium and anticancer drug formulations through this system. Once trapped, the spheroids were protected from shear forces and continuously supplied with oxygen and nutrients by diffusion from the culture medium that circulated through the main channel. Cytokines and cellular byproducts diffused in the opposite direction, away from the spheroids and into the main channel. The authors observed that the spheroid morphology was similar in both the perfused and the static conditions and remained essentially unchanged during a 48-h perfusion period. Remarkably, continuously perfused spheroids exhibited higher chemoresistance to cisplatin, a drug commonly prescribed for MPM patients, when compared with spheroids cultured under static conditions. This simple microfluidic system allowed the use of a small number of cells (about 5000 per assay), which is of paramount importance in personalized medicine scenarios where normally only small-sized biopsies would be available. The possibility of continuous sampling of the supernatant, rather than of the spheroids themselves, enables the use of genomic and proteomic analyses to further interrogate the system.

Huang et al. [21] developed a similar chip, based on the capture and retention of spheroids in semi-circular traps within a microfluidic circuit (Figure 3b). In this system, the authors studied the penetration of nanoparticle carriers into the tumor spheroids. Huang’s microfluidic device consisted of a triple-layer PDMS microfluidic chip composed of four culture chambers (Figure 3b-i,ii), each with five semicircular weirs (each with two apertures to allow perfusion flow) that trapped HepG2 multicellular spheroids (~200 μm) (Figure 3b-iii,iv).

The authors investigated the dynamics of nanoparticle transport around the tumor, adhesion, and internalization/penetration under different experimental conditions. A mass transfer mechanism for nanoparticles penetrating the tumor was proposed based on investigating the effect of several nanoparticle properties (i.e., protein corona, size, and surface charge) on the spatiotemporal performance of nanoparticle-assisted drug delivery. The low penetration and accumulation of NPs in tumors is usually attributed to mechanical or hydraulic resistance. However, the authors’ results suggested that this resistance might have an electrostatic nature, whereby positive charges within the spheroid primarily inhibited the penetration of positively charged NPs (Figure 3b-v). The authors also observed that the protein corona impeded the interaction between cells and NPs (Figure 3b-vi). Overall, this study illustrated an in-vitro contribution to the rational design of NPs for drug-delivery applications at the tumor site using tumor-on-chip systems.

Drug screening within microfluidic platforms is one of the main drivers for spheroid culture. In yet another good example of tumor-on-chip systems based on “naked” spheroids under microflows, Patra et al. cultured human hepatocellular carcinoma cells (HepG2) spheroids of two different sizes in PDMS microfluidic devices (Figure 3c-i) [22]. These tumor on-chip systems featured sets of quadrangular reservoirs or chambers, located at the floor of the microfluidic channel, where the spheroids would be contained. Two different chamber configurations, varying in size (200 × 200 × 250 µm^3^ and 300 × 300 × 250 µm^3^), were tested (Figure 3c-ii). The interior of the PDMS device was treated with oxygen plasma to render the PDMS surface hydrophilic, and Synperonic™ was used to make the device repellent to cell adhesion. A remarkable feature of this tumor-on-chip system was its independence from peristaltic or syringe pumps, as gravity-driven flows (Figure 3c-iii) were used to conveniently perfuse the spheroids under culture in the rectangular chambers. The authors evaluated the effect of cisplatin, a commonly used anticancer agent, in spheroids of different sizes. They also ran similar experiments in 2D conventional culture systems, and they confirmed that the cell culture format (3D spheroid or 2D monolayer) and the size of the spheroid were key determinants in the observed drug responses. Cisplatin had its highest apoptotic activity in small spheroids. Surprisingly, even when treating spheroids with a relative high drug concentration, only 30% (or fewer) tumor cells died in 3D spheroid drug testing. The spheroids in 2D cultures were significantly more susceptible to cisplatin.

Chen et al. [177] developed a 3D breast-cancer-on-chip device aimed at mimicking the transport of NP-based drugs from the blood stream and into a tumor (Figure 3d-i). The device was composed of an endothelial monolayer, ECM, and uniformly sized multicellular type tumor spheroids (MCTS) (Figure 3d-ii–iv). The MCTS, composed of BT549 or T47D cells (two cell lines representative of triple negative breast cancer), were retained within semi-circular traps (Figure 3d-v) and cultured for extended times (14 days) to reach diameters of 150–200 µm (Figure 3d-vi). This microfluidic device was used to monitor the real-time transport of drug-loaded carbon dots (CDs) through the endothelium composed of human umbilical vein endothelial cells (HUVECs) and its penetrability into the MCTS. The CDs were functionalized with PEG and folic acid (FA) and loaded with doxorubicin (DOX), a drug widely used in clinical treatment of breast and other cancer tumors. This microfluidic system enabled real-time monitoring of the transport, efficacy, and cytotoxicity of a nanoparticle-drug system on the same platform.

Lim and Park [23] developed a tumor-on-chip device coupled with a concentration gradient-generator for evaluating the efficacy of cancer drugs in spheroids (Figure 3e-i,ii). This device, made of PDMS, consisted of 50 microwells each 400 µm in diameter and 200-µm deep (Figure 3e-iii). Colon cancer (HCT116) spheroids of approximately 120 µm were cultured in this device and perfused for 3 days in the presence of a gradient of irinotecan, an anti-neoplastic enzyme inhibitor. This tumor-on-chip system illustrated an important microfluidic feature for cancer research, namely the ability to generate gradients (Figure 3e-iv). When applied to personalized medicine approaches, this simple device could represent a convenient tool for the parallel assay of the effectiveness (and safety) of cancer drugs and the determination of the proper dose of the drug in specific patients. Similarly, Mulholland et al. introduced a PDMS microfluidic platform, fabricated through standard microfabrication techniques (soft- and photo-lithography), composed of two units bonded together [178]. This platform allowed the development of reproducible concentration gradients of anticancer drugs, and replicates for each concentration, across an array of multiple-sized spheroids without resorting to external fluid actuation. The authors conducted drug screening experiments using cisplatin, docetaxel, and enzalutamide on human high-grade glioma (UVW) cells and spheroids, and prostate cancer LNCaP cells and spheroids, as well as primary prostate cancer cells. Tumor-on-chip systems with gradient-generators could be effective means to conduct in vitro anticancer drug testing in spheroids derived from tumor biopsies, potentially allowing extensive in vitro drug screening for a more personalized and effective patient treatment. Overall, these platforms could be useful in drug development and personalized medicine, with an evident potential to provide a significant time and cost reduction in the delivery of convenient healthcare to cancer patients.

### 5.2. Spheroids in a Hydrogel Matrix

Various tumor-on-chip systems recreate the confinement of tumorous tissues in an extra-tumoral matrix environment [27,28,29,30,31,32]. In general, in these tumor-on-chip systems, spheroids are embedded within a hydrogel matrix, such as collagen [28,30,31,179], Matrigel [27,32], a gelatin-based hydrogel [29,135], or alginate [119]. In these systems, nutrients and oxygen permeate by diffusion into tumor organoids from channels that emulate capillary circuits. Some illustrative examples of these tumor-on-chip devices follow.

**Figure 4:**
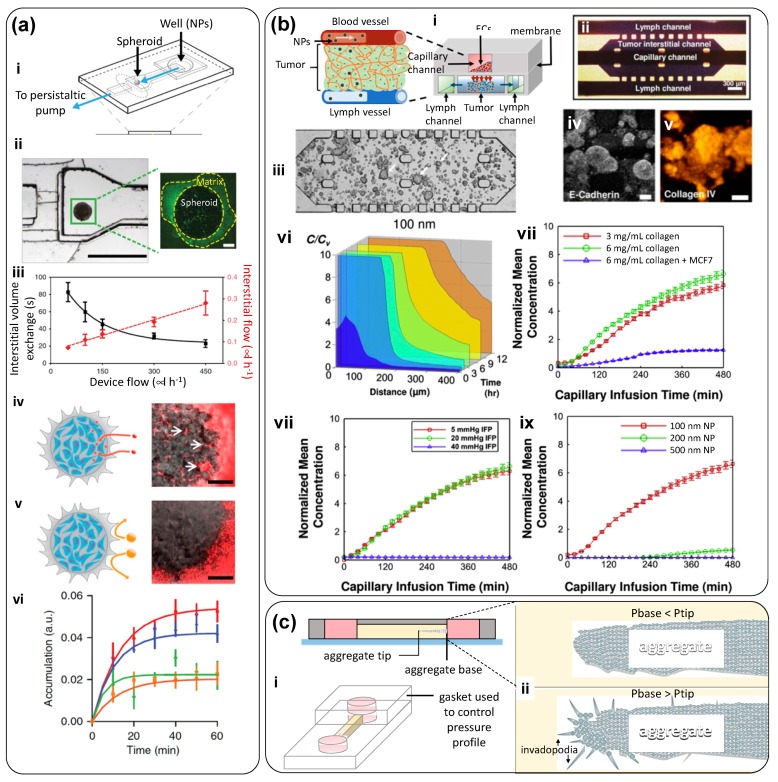
Tumor-on-chip in which a spheroid is embedded in a hydrogel. (**a**) Microfluidic system proposed by Albanese et al. [27]: (i) scheme of the optically accessible TOC, (ii) actual image of the spheroid within the microfluidic chamber (scale bar: 1000 μm). The tumor spheroid is lightly embedded in collagen. Scale bar: 100 μm. (iii) Interstitial volume exchange (residence time within a spheroid) as a function of the inlet flow rate (black squares); the corresponding interstitial flow rate is shown (red diamonds). (iv) Scheme and image of 40 nm PEG nanoparticles penetrating the spheroid and accumulating in the interstitial spaces (scale bar: 100 μm). (v) Larger nanoparticles (d = 110 nm) cannot penetrate the spheroid. Scale bar: 100 µm. (vi) Nanoparticle of different sizes [40 nm (red); 70 nm (blue); 110 (green), and 150 (blue)] exhibit different accumulation rates within spheroids. Nanoparticles were injected into the chamber at 50 mL/h; (**b**) The tumor on chip designed by Kwak et al. [28] aimed to recapitulate (i) the dynamics induced by the pressure gradient between the capillary and the lymphatic nets. (ii) Scheme (scale bar: 300 μm) and (iii) actual microdevice: cancer spheroids (indicated by arrows) develop within collagen in the central compartment by day 3. These spheroids, like actual tumors, express (iv) E-cadherin and (v) collagen IV. Scale bar: 50 µm. This system enables studies of accumulation of nanoparticles within tumors. (vi) Concentration of 100 nm particles at different distances of the capillary boundary (vii) Accumulation of nanoparticles as a function of matrix stiffness, (viii) interstitial pressure, and (ix) particles size. Taken from reference [28]; (**c**) TOC devised by Piotrowsky-Daspit [30] to study the effect of intra-tumor interstitial pressure on tumor invasiveness. (i) Schematic representation of the device; (ii) Micrographs showing an E-cadherin-GFP aggregate under a condition where the pressure gradient favors invasiveness (higher pressure at the tumor core). Modified from Piotrowsky-Daspit [30].

Albanese et al. studied real-time NP transport and NP accumulation into tumor spheroids embedded in a microfluidic chamber (Figure 4a) and simulated physiologically relevant flow conditions within the optically accessible device [27]. The authors developed a tumor-on-chip consisted of a PDMS microfluidic device composed of an inlet channel and a visualization chamber (Figure 4a-i). Nanoparticles were fed at the inlet chamber. Tumor spheroids 280 µm in size were immobilized inside the visualization chamber by slight compression against a glass cover-slip. The channel entrance (600 µm wide) opened further (1200 µm) at the visualization chamber to reduce the linear velocity of the fluid and yield a gentle motion around the spheroids to minimize physical damage (Figure 4a-ii). The authors recreated different dynamic scenarios by varying the inlet flow rate. For instance, they determined the average time for medium exchange within the spheroids—a sort of spheroid residence time (the amount of time required for the non-fluorescent medium inside the spheroid to be replaced by a fluorescent medium) (Figure 4a-iii). The penetration of NPs into the tissue was mainly influenced by their diameter (Figure 4a-iv–vi) and NP transport was predominantly dispersion-limited. For larger particles (i.e., larger than 100 µm), the inlet flow rate affected their accumulation exclusively at the spheroid perimeter and did not increase their penetration depth (Figure 4a-v,vi). However, retention could be improved by specific receptor-targeting. The authors also conducted experiments in animal models to validate these observations. Their results showed that this tumor-on-chip can be used for screening NP designs prior to in vivo studies.

Kwak et al. developed a tumor-on-chip system to study the transport of drug-loaded NPs into tumor spheroids under different (and well controlled) gradients of pressure [28] (Figure 4b). This microfluidic system had a sophisticated system that allowed precise tuning of the transport conditions within the device. It was made by sandwiching a porous membrane between two layers of microchannels (Figure 4b-i). The top layer had a channel that emulated a capillary. The endothelial layer of the capillary was mimicked by culturing human microvascular endothelial cells over a porous membrane coated with Matrigel. The bottom layer had a middle chamber and two lateral channels (Figure 4b-ii). Lines of periodically placed posts served as divisors between the main chamber and the lateral channels. The central chamber represented the 3D tumor tissue. An MCF7 cell-laden collagen suspension was placed within the central chamber. Various cell concentrations (in the range of 10^6^ to 10^8^ cells/mL) and collagen solutions (3 and 6 mg/mL) were tested (Figure 4b-iii). After a few days, tumor spheroids grew within the central chamber and exhibited typical tumor biomarkers (Figure 4b-iv,v). An interstitial channel allowed for the application of pressure to displace liquid throughout the tumor chamber, while the side channels simulated lymphatic conduits.

The authors established different pressure gradients between the capillary, the interstitial space, and the lymphatic channels to simulate different dynamic environments (Figure 4b-vi–ix). This was done simply by varying the column heights of the culture medium reservoirs that fed each of the six inlets. Pressures between 40 and 5 mmHg were applied at both ends of the interstitial channel, and a pressure of 5 mmHg was applied at the inlet ports of the four lymphatic channels. Consistent with the physiological conditions, a capillary pressure of approximately 20 mmHg was applied. A small pressure differential was also established between both ends of the capillary channel (i.e., 20 mmHg to 19.25 mmHg) to facilitate the transport of NPs along the capillary, yielding an average capillary fluid velocity of ~0.3 mm/s. The NPs were transported through this 3D tissue structure and reached the cancer cells. The authors studied the effect of different parameters on the transport and uptake of nanoparticles by tumors including cut-off pore size and interstitial fluid pressure. The results suggested that this tumor-on-chip device emulated the complex dynamics around the tumor, yielding detailed information about NP transport within a tumor niche. This finding highlights the argument that NPs should be designed based on their dynamic interactions with tumor microenvironment. A variation of this system was later used by Shin et al. [29]. The authors developed a simple tumor-on-chip by placing breast tumor spheroids, embedded in Matrigel, in the central chamber of a microfluidic device. Liquid medium was continuously fed at 0.5 µL/min into each of the two lateral channels that flanked the central spheroid-laden compartment. The authors fabricated uniformly sized spheroids by first seeding MCF7 cells into an array of wells imprinted in Matrigel, and then incubating for 14 days. They produced this array using a PDMS master template containing circular posts 50 μm in diameter and 30 μm in height.

In yet another example of spheroid system embedded in hydrogels, Piotrowsky-Daspit et al. used a tumor-on-chip system (Figure 4c-i) to examine the role of high intratumoral interstitial pressure in triggering a biochemical cascade of events that favors cancer cell-invasion [30]. The authors engineered a simple PDMS device, consisting of two cylindrical ports and an interconnecting channel. Collagen I was deposited in the channel, and a needle was used to fabricate a blunt cavity from one of the ports. Then, breast cancer cells (MDA-MB-231) or prostate cancer cells (PC3) were cultured within a collagen matrix to form elongated aggregates embedded in the collagen matrix. By this ingenious fabrication technique, the base of the elongated tumor (the base) remained in direct contact with one of the ports while the tip was embedded within the hydrogel, and a differential pressure could be established between the base and the tip of the tumor by setting different liquid column heights (culture medium) among the ports. The authors observed that even simple pressure gradients were able to induce distinct invasiveness behaviors. For example, when P_base_ was higher than P_tip_, an invasive phenotype was favored (Figure 4c-ii).

### 5.3. Non-Spheroid Models where 3D Cancer Tissues are in Contact with Non-Cancerous Tissues

In previous sections, we have reviewed examples of tumor-on-chip systems in which the behavior of solid tumors is recreated in the absence of healthy tissue. These systems can recapitulate many of the relevant aspects of solid tumors; however, tumors exist in a multi-cellular environment. In addition, in physiological situations, tumors are surrounded by non-cancerous tissues. The interplay between cancerous and “healthy” tissues is both relevant for tumor progression and challenging to study using animal models. Therefore, the presence of multiple cell types is key for a precise recreation of aspects of the tumor microenvironment related to the crosstalk between different cell populations. Tumor-on-chip systems offer the possibility of recreating, *in vitro*, some of the complex in vivo conditions in terms of cellular constituents, signals, and factors that contribute to tumor malignance, cancer growth, treatment efficacy and toxicity, and metastasis. However, the proposition of adding normal cells to a tumor-on-chip device is challenging and imposes a higher level of sophistication on the design, fabrication, and operation of these devices.

**Figure 5:**
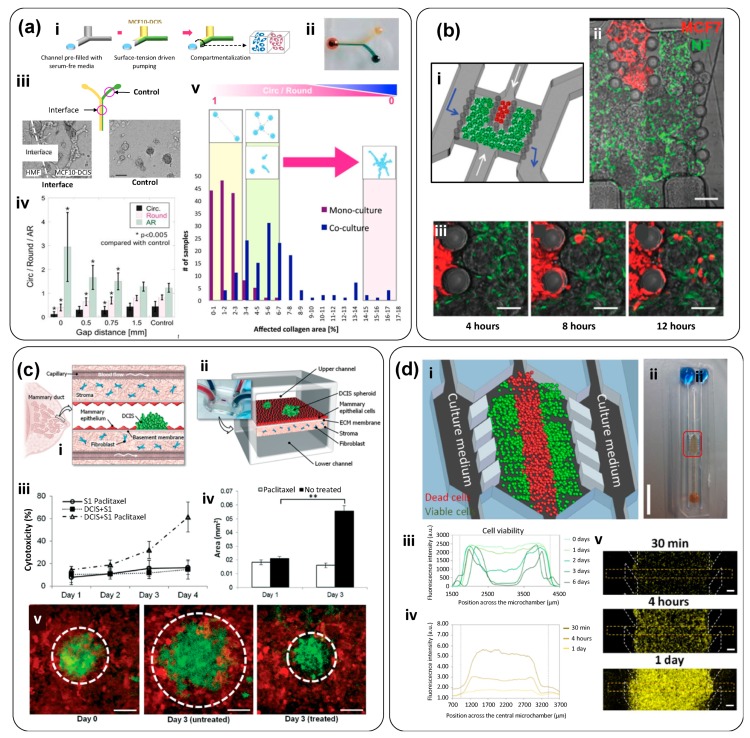
Tumor-on-chip (TOC) systems in which cancerous and normal microtissues share an interface. (**a**) Crosstalk between cancer cells and fibroblasts in a Y-shaped TOC: (i) scheme and (ii) actual device. (iii) Human mammary fibroblasts (HMF) and breast cancer (MCF10-DCIS) cells establish a cross-talk at the interface between the cancerous and normal microtissue sections. Scale bar: 30 µm. (iv) Circularity, roundness, and aspect ratio (AR) of MCF10-DCIS cancer cells as a function of their distance from the interface. (v) Image analysis characterization of invasiveness. Taken from reference [33]; (**b**) On-chip activation of stromal tissue by crosstalk with cancerous tissue [35]: (i) schematic representation of the device and (ii) actual micrographs of normal cells (expressing GFP) and cancer cells (expressing RFP). Scale bar: 100 μm. (iii) Time sequence showing cancer cell migration and invasion. Scale bars: 100 μm; (**c**) TOC system that recapitulates early stage breast cancer: (i) tumor-niche aimed. (ii) Schematic representation of the TOC system. (iii) Cytotoxicity against cancerous DCIS spheroids and normal epithelial cells at different feeding conditions: continuous administration of Paclitaxel through the lower channel (open triangles), batch administration of paclitaxel on normal epithelial cells (open circles), and negative control (squares). (iv) Change in the projected area of a DCIS spheroid treated with paclitaxel in the TOC (white bars) and untreated (black bars; ** *p* < 0.05) (v) Micrographs of DCIS spheroids at day 0, after three days of culture, and after three days of paclitaxel treatment. Scale bars: 100 μm; (**d**) TOC by Ayuso et al. [36] to recapitulate hypoxia and nutrient gradients within cancerous tissue: (i) schematic representation and (ii) actual device. Scale bar: 1 cm. (iii) Evolution of the cancer cell viability profiles, and (iv-v) hypoxia levels within the device. Scale bar: 400 μm Taken from reference [36].

The elucidation of the complex paracrine signaling that is implicated in cancer pathogenesis is one of the aspects needing study in tumor-on-chip models that also incorporate normal cells. Sung et al. developed a simple tumor-on-chip to study a key step in breast cancer progression, namely the transition of ductal carcinoma in situ (DCIS) into invasive ductal carcinoma (IDC) [33]. The authors designed a simple compartmentalized microfluidic device in which non-cancerous human mammary fibroblasts (HMFs) were co-cultured with mammary epithelial cells (MCF-DCIS). This Y-shaped device (two inputs and one output) allowed sample loading using surface-tension–driven pumping (Figure 5a-i,ii). Cell loading of normal and cancerous cells into this device was possible either simultaneously or sequentially. A mix of Matrigel and collagen I (1:1) was used as the loading matrix. The HMFs and MCF10-DCIS cells established a crosstalk at the interface between the cancerous and normal microtissue sections. MCF10-DCIS cancer cells adopted different shapes (i.e., their circularity, roundness, and aspect ratio significantly changed) as a function of their distance from the interface (Figure 5a-iii). Clusters of cells in co-culture (HMF and MCF10-DCIS) affected the structure (physical appearance under the microscope) of the matrix significantly more than did monocultured MCF10-DCIS cells. This effect was quantified by image analysis (Figure 5a-iv).

The change in shape of MCF710-DCIS cells, and their ability to remodel the matrix, suggested the onset of a DCIS to IDS transition. This transition strongly depended on the distance between the cells, being more evident around the cell interfaces. Sung’s experimental results suggested an underlying two-step molecular mechanism for the transition from DCIS to IDS (Figure 5a) [33]. An initial morphological change was first observed when HMFs were cultured at some distance (0.5–1.5 mm) from the MCF-DCIS cells, suggesting that soluble factors could initiate the transition, and the cell–cell contact between HMFs and MCF-DCIS cells favored the completed transition to an invasive phenotype. This is simple and clever tumor-on-chip device can be easily adapted to study a vast range of signaling phenomena.

Gioiela et al. developed a microfluidic tumor-on-chip system to replicate the changes that occur during the invasion of malignant epithelial breast cancer into healthy stroma [35]. Their optically accessible microfluidic chip enabled the culture of three-dimensional microtissue precursors (3D-µTP) under dynamic flow conditions (Figure 5b). This tumor-on-chip was composed of two culture chambers (Figure 5b-i). Stroma cells were cultured in the outer chamber, and then breast cancer cells were placed and cultured in the inner chamber. The chambers were separated by arrays of regularly spaced posts and both had a dedicated channel for tumor and stromal loading and two additional side channels that allowed culture medium flow. Three different cell lines were used to fabricate the 3D-µTP. Normal fibroblasts (NF), cancer-activated fibroblasts (CAF), and human breast adenocarcinoma cells (MCF7) were used to produce NF-µTP, CAF-µTP, and MCF7, respectively. Cells were transfected using a viral vector to induce expression of green and red fluorescent proteins (Figure 5b-ii). The authors used a syringe pump to feed culture medium into these devices at a nominal flow rate of 3.0 µL/min and performed immunofluorescence assays and optical microscopy directly within the microfluidic system. The intrinsic fluorescence of the 3D-µTPs enabled the monitoring, in real time, of tumor cell invasion in the adjacent normal-cell chamber. The authors demonstrated the activation of NF into myofibroblasts induced by the co-culture. The generation of “activated” stromal tissue was characterized throughout α-smoot muscle actin and platelet derived growth factor (PDGF) immunofluorescence evaluation.

In addition, the experiments conducted in this microfluidic system enabled the evaluation of changes in fibronectin, hyaluronic acid, and collagen expression/production. An increase in hyaluronic acid, fibronectin, and collagen contents, and a fine-to-coarse transition of the arrangement of the collagen network, was observed in the “activated” stromal tissue. Thus, this tumor-on-chip offered the possibility of characterizing stromal cell activation in the presence of cancer cells. In general, this tumor-on-chip platform was confirmed to be an effective tool for studying the interactions between malignant and normal cells [35].

Choi et al. co-cultured breast spheroids (MCF10-DCIS), HMFs, and human mammary epithelial cells (HMT-3522; S1) embedded in a collagen type I-hydrogel to replicate the microarchitecture of DCIS in a tumor-on-chip (Figure 5c-i) [34]. The device was composed of two chambers separated by a collagen-derived membrane (Figure 5c-ii). In the upper chamber, DCIS spheroids of approximately 150 μm in size were embedded in the epithelium to recreate the ductal lumen and allow the continuous flow of culture medium. The fibroblast-coated stromal layer in the lower chamber simulated the capillaries of the mammary stroma. Cell proliferation was observed by engineering the epithelial mammary cells, the mammary fibroblasts, and the MCF10-DCIS to express red fluorescent protein (RFP), cyan fluorescent protein (CFP), and green fluorescent protein (GFP), respectively. Anticancer drug exposure experiments were conducted using paclitaxel, a commercial anticancer drug, at a concentration of 20 nM (Figure 5c-iii,iv). This model system enabled the establishment of physiologically relevant tissue architectures, the location of micro-environmental cues, and a direct observation of cell responses.

In yet another example, Ayuso et al. developed a polystyrene-based microfluidic device to study a tumor microenvironment in real time (Figure 5d) [36]. Their system enabled the real-time measurement of the concentration of reactive oxygen species (ROS), as well as the assessment of cell proliferation and apoptosis as a response to different oxygen, glucose, and anticancer drugs concentrations. Indeed, the configuration of the central chamber allowed the spontaneous generation of normoxic, hypoxic, and necrotic regions within the device. This tumor-on-chip consisted of a central microchamber flanked by two lateral microchannels. Tumor cells were embedded within a collagen matrix in the central microchamber (Figure 5d-i,ii), while the lateral microchannels were used to perfuse medium, oxygen, natural killer (NK) cells, and anticancer drugs. In different sets of experiments, the authors cultured two cancer cell lines—glioblastoma U-251 MG cells and HCT-116 from colon carcinoma—in this tumor-on-chip system. They demonstrated the existence of a necrotic core in the middle portion of the central chamber, suggesting the development of gradients of nutrients and oxygen (Figure 5d-iii). Experiments using a fluorescent dye responsive to low oxygen concentrations further demonstrated the establishment of steep oxygen gradients within the middle chamber (Figure 5d-iv,v). The authors also modeled the effect of two different anticancer agents, DOX for colon carcinoma and temozolomide (TMZ) for glioblastoma, and showed the interplay between hypoxia levels and therapeutic effects. For instance, perfusion of DOX at a dose of 30 µM through the lateral microchannels significantly reduced the number of viable HCT-116 cancer cells in the hypoxic core. These experiments suggested a correlation between differences in the cytotoxic response of tumor cells and differences in the proliferative rate of cells within different regions of the tumor-on-chip. Due to its configuration, this device enabled the establishment of clear oxygen gradients and hypoxic conditions and the study of anticancer drugs in cell populations under controlled oxygen gradients, without resorting to the use of spheroids.

### 5.4. Spheroids Surrounded by “Healthy” Tissues

**Figure 6:**
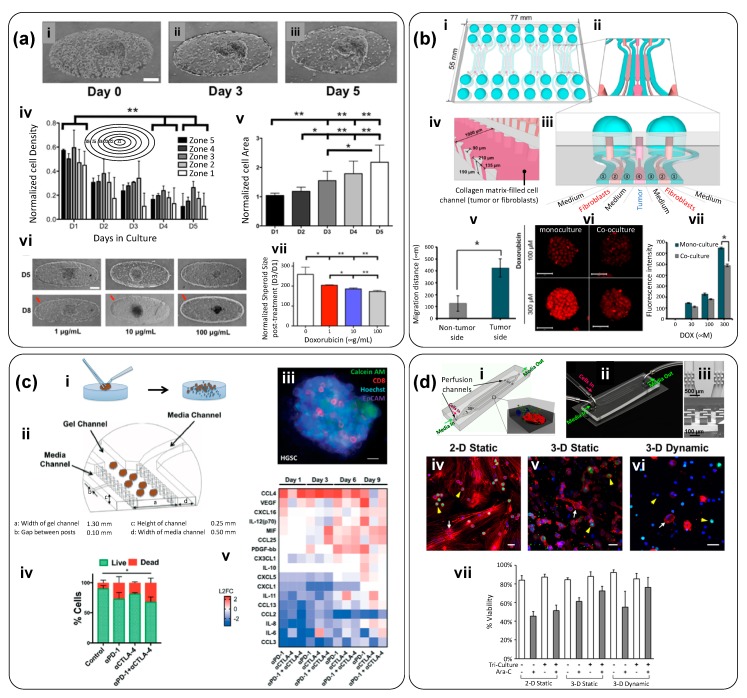
Tumor-on-chip (TOC) systems that consider spheroids embedded in non-cancerous cell-laden-hydrogels. (**a**) Chemotaxis-driven assembly of endothelial barrier in a TOC platform [50]. Brightfield images of MCF7 spheroids co-cultured with human umbilical vein endothelial cells (HUVECs) (i) immediately after encapsulation, (ii) after three, and (iii) five days of culture. Scale bars: 200 μm. (iv) Evolution of HUVEC densities within different GelMA hydrogel zones 1 through 5 (zones are indicated on the oval inset). Zones are indicated within the inset. (v) Spheroid size as a function of culture time. Size was quantified as the projected area of the spheroid and normalized to its size at day 0; (*) and (**) indicate statistically significant differences of *p* < 0.05 and 0.01, respectively in a pairwise *t*-test. (vi) HUVECs and MCF7 spheroids (as observed in bright field) five (D5) and eight days (D8) after doxorubicin (DOX) treatment. Red arrows indicate the presence (or absence) of endothelial barrier. Scale Bar: 200 μm. (vii) MCF7 spheroid size after treatment with increasing doses of DOX. The spheroid area was normalized with respect to that of untreated spheroids. Significant differences are indicated by (*, **): *p* < 0.05 and 0.01, respectively (pair wise *t*-test). Taken from reference [50]; (**b**) TOC developed by Jeong et al. [51] to study the crosstalk and mutual activation between fibroblasts and cancer cells: (i) schematic of the device, (ii) and (iii) successive close-ups on the channel section, (iv) detail of the cell culture channel, and (iv) comparison of migration distances of fibroblasts in the direction of the cancerous region (blue) and the non-cancerous region (gray). (vi) Micrographs of cancer spheroids treated with different doses of DOX in monoculture and co-culture conditions. Scale bars: 50 μm. (vii) Evaluation of DOX efficacy in co-culture and monoculture conditions in this TOC. Significant differences are indicated by (*): *p* < 0.05. Taken from reference [51]; (**c**) TOC developed by Aref et al. [48], to model immune checkpoint blockade. (i) A real tumor is subjected to dissociation (mechanical and enzymatic) to yield dissociated tumor tissue (spheroids, cell agglomerates and single cells); (ii) schematic of Aref’s TOC; (iii) spheroid stained to reveal the presence of calcein AM (green); CD8 T cells (red); tumor cells (EpCAM; purple); and all nucleated cells (Hoechst; blue). Scale bars: 20 μm. (iv) Cell viability, and (v) cytokine expression profile over time in spheroids derived from patients and treated with different immunotherapies: α-PD-1: pembrolizumab, 250 μg mL^−1^); α -CTLA-4: (ipilimumab, 50 μg mL^−1^); or a combination. Taken from reference [48]. (**d**) TOC proposed by Bruce et al. [49] to recreate a bone marrow microenvironment and study acute lymphoblastic leukemia (ALL): (i) schematic representation, (ii) actual image, (iii) and detail of the post array within the device. (iv–vii) Confocal images of co-cultures of SUP-B15 (yellow arrows) and BMSC cells (white arrows) in (iv) 2D static, (v) 3D static, and (scale bars: 500 μm and 100 μm) (vi) 3D dynamic models. Proliferating cell nuclei were stained with Ki67 (green). Actin filaments were stained with phalloidin (red). Cell nuclei were stained with DAPI (blue). Scale bars: 20 μm. (vii) Chemoresistance of tumor cells to Ara-C among tri-culture or monoculture systems, in 2D versus 3D culture, and exposed (or not) to interstitial flow. (+) and (*) indicate significant difference between groups of *p* < 0.1, and *p* < 0.05, respectively. Taken from reference [49].

In some instances, tumor spheroids have been embedded in hydrogels containing normal (i.e., non-cancerous cells) within microfluidic chambers. For example, Aung et al. [50] developed a tumorous microtissue encapsulated by an endothelial barrier composed of HUVECs. The authors mixed breast cancer spheroids (MCF-7) into a suspension of HUVECs (~50 spheroids into 5 mL of a HUVEC suspension containing 0.4 × 105 cells/mL). This mix was centrifuged, and the pellet was suspended in 100 µL of 10% gelatin methacryloyl (GelMA); this new mixture was then loaded into thin microfluidic devices. Using a microscope, the authors located areas where cancer spheroids were surrounded by HUVECs and photocrosslinked these locations, flushing out the rest of the non-crosslinked hydrogel. This operation resulted in a hydrogel micro-cosmos containing spheroids surrounded by HUVEC cells. Over time, the HUVECs chemotactically migrated from the bulk of the hydrogel towards its perimeter and spontaneously developed an endothelial barrier around the GelMA construct (Figure 6a-i–iii). As a result, starting from homogeneity, the HUVEC density varied in different areas within the constructs over time (Figure 6a-iv). The size of the tumor spheroids, as determined by their projected area, also increased over time (Figure 6a-v). Thus, this tumor-on-chip produces tumor spheroids within a semisynthetic extracellular matrix embedded in a cell-monolayer endothelial barrier conformed by tight cell-cell E-cadherin junctions and encompassing the entire length of the GelMA hydrogel. This simple and smart platform was also used to evaluate the effect of DOX (Figure 6a-vi,vii).

After 5 days of cultivation, they administered different doses of DOX (1, 10 and 100 µg/mL) for 3 days. Figure 6a-vi shows bright-field images of the HUVEC-MCF7 constructs before (top row) and 3 days after (bottom row) DOX treatment. Red arrows indicate the endothelial barrier. At concentrations of 10 and 100 μg/mL, DOX destroyed the endothelial barrier, decreased the size of spheroids, and caused a darkening of the MCF7 spheroids (Figure 6a-vii).

Jeong et al. developed a tumor-on-chip to investigate the 3D interplay between cancer cells and fibroblasts (Figure 6b) [51]. This tumor-on-chip system consisted of four units; each unit comprised seven channels, each one 1000 μm wide and 190 μm deep (Figure 6b-i–iv). Channels 2, 6, and 4 were designated for non-cancer or cancer cell culture, and channels 1, 3, 5, and 7 were used for medium filling (Figure 6b-iii). The device was designed for intermittent feeding and the cell culture medium was changed every day. A collagen I solution (2 mg/mL) was mixed with the cells before being loaded into the device channels. Mono- and co-cultures of HT29 cancer cells and normal fibroblasts (CCD-18Co) were performed (Figure 6b-v) and, in some experiments, naïve collagen I (without cells) was loaded in the different channels of the device. A crosstalk between the cancer cells and fibroblasts was observed. After 5 days, the HT-29 cells formed viable spheroids and their growth was stimulated by co-culture with fibroblasts (Figure 6b-v). Increased expression of α-SMA and enhanced migration activity suggested fibroblast activation. The authors also observed changes in the expression of intracellular proteins related to angiogenesis and apoptosis. For instance, CD26, GM-CSF, SerpinE1, TIMP-1, HB-EGF, TSP-1, and GDNF were up-regulated and phospho-p53 (S15), phospho-p53 (S46), phospho-p53 (S392), pro-caspase-3, and cytochrome C were down-regulated in the tumor spheroids in co-culture. The spheroids co-cultured with fibroblasts exhibited increased expression of fibronectin, along with reduced expression of Ki-67. The effects of DOX and paclitaxel were also evaluated in this platform. Co-cultured spheroids also exhibited lower levels of DOX uptake and reduced sensitivity to paclitaxel (Figure 6b-vii). This 3D co-culture platform enabled the study of tumor microenvironment factors related to the epithelial-mesenchymal transition process, fibroblast activation, and drug resistance.

Aref et al. conducted experiments in a commercial DAX-1 microfluidic system (Aim Biotech) in which patient-derived spheroids (PDOSTS) were cultured in contact with immune system cells to recreate the tumor immune microenvironment and screen the response of tumors to immunotherapy (therapies based on antibodies) [48]. Tumor biopsies from patients were mechanically and enzymatically dissociated to yield spheroids, cell agglomerates, and single cells (Figure 6c-i). The spheroids were separated for culture in the DAX-1 microfluidic device (Figure 6b). The DAX-1 chip was composed of three microfluidic chambers—a central gel channel surrounded by two channels for media circulation (Figure 6c-ii). The PDOSTS spheroids (40–100 μm in diameter) were embedded in collagen type I hydrogels in the central chamber and cultured. In the side channels, medium with or without monoclonal antibodies was circulated. The authors demonstrated the presence of autologous tumor-infiltrating immune cells (CD8-T) (Figure 6c-iii). Treatment with an anti-PD-1 antibody showed cancer cell death mediated by CD8 T-cells. Analysis of cytokines (Figure 6c-iv,v) revealed a natural evolution of cytokine and growth factor secretion over time (e.g., IL-8 VEGF, IL-12, CCL4). This study demonstrated that patient-derived tumor spheroids retain lymphoid and myeloid subsets of immune cells and that those cell populations could be further cultured in microfluidic chips.

Bruce et al. studied acute lymphoblastic leukemia (ALL) in a 3D microfluidic system (Figure 6d) [49]. The device consisted of four perfusion microchannels. Chip dimensions were set considering the nutrient diffusion limits (i.e., relevant diffusional length scales) and aimed to recreate the interplay between the different cell types involved in ALL (Figure 6d-i,ii). Cell loading and culture medium feeding was performed through two ports (“cell in” and “media in”) placed at the inlet side of the device. SUP-B15 leukemic cells were cultured in the presence or absence of human bone marrow stromal cells (BMSCs) and human osteoblasts (HOBs). Cells were suspended in medium with 88% collagen type I—a concentration that is consistent with the stiffness of bone marrow (< 300 Pa). After collagen I gelation, culture medium was fed through the inlet and into the microchannels. Velocity profiles within the microchannels, set to be similar to that in the interstitial space of the bone marrow, were maintained at 0.27 ± 0.18 μm/s. Cell culture was performed in 2D static, 3D static, and 3D microfluidic models (Figure 6d-iv–vi), and significant differences in cell morphology and expression were found. The authors used this microfluidic platform to evaluate cell-cell and cell-matrix interplay in a controlled microenvironment that recapitulated physiological ranges of critical dynamical variables. Indeed, the 3D static and microfluidic models demonstrated coordinated cell-cell and cell-matrix interactions that were not detectable in the 2D static model. The 3D platform proved to be a more accurate bone marrow niche model, where different cells in culture (leukemic cells, human bone marrow stromal cells, and human osteoblasts) could interact in a dynamic fashion. In addition, chemoresistance studies were carried out by exposing 2D and 3D cultures to cytarabine (Ara-C), and the bone marrow microenvironment was found to have a protective role in cancer cell survival during treatment. Moreover, a lower chemotherapeutic sensitivity of leukemic cells was observed in the 3D tri-culture models than in the 2D models (Figure 6d-vii,viii). The 3D microfluidic model developed in this work is a suitable in vitro option for the study of tumor cell biology in the bone marrow niche (i.e., the space where the leukemic disease is initiated) and the site of metastatic malignancies characterized by high anticancer drug resistance.

### 5.5. Vascularized Tumor-On-Chip Systems

The presence of vascularization is one of the landmarks of tumor environments [57,180]. Therefore, vascularization is another relevant aspect to consider when engineering a tumor local microenvironment [180,181]. Indeed, vascular transport is particularly required for tissues with diffusional distances greater than 200 μm [8,182,183], and tumors are not an exception. When a tumor exceeds a critical diameter of 200 μm, hypoxia develops at its central core, and a complex biochemical cascade commands vascularization to start [126,184]. A faithful recapitulation of the physiology of solid tumors greater than 200 μm demands vascularization to be engineered around or within the tumor.

**Figure 7:**
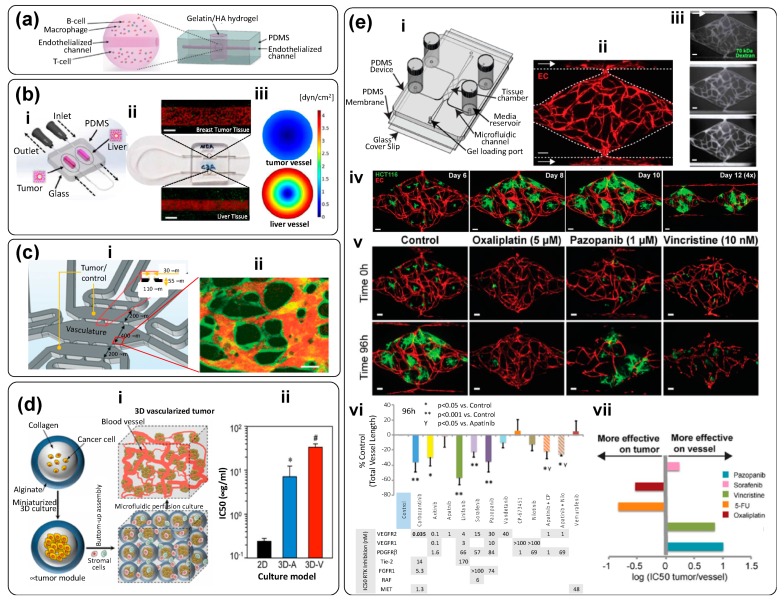
Vascularized tumor-on-chip (TOC) systems. (**a**) A gelatin and hyaluronic acid (HA) construct, built in a PDMS device, traversed by an endothelialized microchannel. Taken from reference [54]; (**b**) TOC devised by Ozkan et al. [55]. (i) Schematic representation of a breast tumor microenvironment connected to a healthy liver niche. (ii) Close-up view of the TOC system. Confocal images show GFP-breast cancer cells (green) and particles circulating inside the vascular channel (red) and FITC-anti-albumin immunostained healthy liver cells (green) and particles circulating inside the vascular channel (red). Scale bar: 500 µm. (iii) CFD simulation showing the cross-sectional shear stress profiles in tumor and healthy vessels. Taken from reference [55]; (**c**) TOC by Shirure et al. [56]. (i) Schematic representation of the device, and (ii) confocal slice of the microvascular network (green) fabricated within the TOC central chamber and perfused with fluorescently labeled dextran (orange). Scale bar: 50 µm. Taken from reference [56]; (**d**) Bottom-up engineering of vascularized tumor models proposed in Agarwal et al. [57]. (i) Schematic representation of the strategy. (ii) Comparison of the effect of docetaxel, expressed as IC50, for three different microtumor models: 2D (black), a-vascular 3D (blue) and vascularized 3D (red) models; (*) indicate statistically significant difference of *p* < 0.05 when 3D vascularized models are compared to 2D culture model, and (#) significant differences of *p* < 0.05 when 3D vascularized models are compared with 2D and 3D avascular culture models. Taken from reference [57]. (**e**) Vascularized tumor-on-chip developed by Sobrino et al. [58]. (i) Scheme of the microfluidic device: a PDMS microfluidic chip composed of an array of three tissue chambers, each connected to two straight channels mimicking an arteriole (line of higher pressure) and the venule (line of lower pressure). Channels were also connected to the medium inlets and outlets. A hydrostatic pressure differential of 10 mm H_2_O was established to enable flow across the microfluidic channel, while a pressure gradient of 5 mm H_2_O was set across the tissue chambers to favor transport across the hydrogel. (ii) A human-endothelial cell vasculature net was fabricated within diamond-shaped tissue chambers containing EC-like matrix. Remarkably, the development of vasculature in these tissue chambers occurred spontaneously by self-assembly of EC cells initially seeded within the channels and the tissue chamber at day zero. Endothelial cells were continuously transduced with mCherry for easy visualization of the network (iii) Experiments of continuous perfusion of Dextran X show that the vascular network is practically leak-free after several days. (iv) Growth of tumors, fabricated by a spheroid formation method assisted by thrombin, seeded within the vasculature network models after 6, 8, 10, and 12 days of culture at continuous perfusion. (v) Different drugs were tested in this tumor-on-chip system; (vi and vii) the model correctly discriminates between drugs that target the tumor-vasculature formation capabilities and those that target the tumor. Scale bars: 50 µm. Taken from reference [58].

Engineering vascularization is not a trivial task. Fortunately, the recent literature contains good examples of vascularized tumor-on-chip systems [54,56,57,58,60]. For example, Mannino et al. [54] fabricated a continuously perfusable system with a central vessel of approximately 350 µm to recreate an in vitro B-cell lymphoma tumor model (Figure 7a). This lymphoma-on-chip model consists of a HyStem-C hydrogel-based tumor traversed by a round and perfusable vascular channel. The system recapitulated the interactions between cancer, endothelial, and immune cells.

In another study [54], A20 cell line mouse B-cell lymphoma was used to replicate diffuse large B-cell lymphoma (DLBCL), and mouse lung microvascular endothelial cells (MLMVECs) were used as endothelial cells. In this easy-to-build device, the authors employed common laboratory materials and easy fabrication techniques. For example, they used a stainless-steel wire to create the vascular microchannel (internal diameter of 328 ± 51 µm). Using this channel, medium was perfused at a rate of 2 µL/min. Notably, this simple tumor-on-chip was adequate for testing the efficacy of an immunotherapy drug. An antibody was incorporated into the medium to block the colony-stimulating factor 1 receptor (CSF-1R), causing macrophage cell death. Due to its simplicity, this model will enable future experiments with different tumors. Overall, Mannino et al. demonstrated a simple strategy that enables researchers to recreate tumor microenvironments using resources readily available in most labs [54].

Ozkan et al. aimed at a partial recreation of the interplay between healthy or tumorigenic liver and breast tumor niches throughout the development of a simple dual organ-on-chip [55]. The system connected a liver microtissue and a breast cancer tumor module via an endothelialized vascular channel (Figure 7b-i). The authors used healthy liver cells (THLE-3), carcinoma liver cells (C3Asub28), and human breast cancer cells (MDA-MB-231). Their PDMS tumor-on-chip device (casted in an aluminum mold; Figure 7b-i) was bonded to a glass slide (i.e., it was optically accessible), and both organ compartments (liver and tumor) were filled with a suspension of collagen and cells. Collagen concentrations of 7 mg/mL and 4 mg/mL were used in the case of carcinomas and healthy tissues, respectively. These concentrations were set to match the compression modulus of human tumors and liver tissues. Notably, the fabrication procedure was ingenuous and simple. Needles were inserted into the cavities to fabricate the channel that simulated the central vessel. Once the collagen was polymerized, the needles were removed, and telomerase immortalized microvascular endothelial (TIME) cells were seeded and incubated within the channel for 72 h to endothelialize the vessel (Figure 7b-iii). Computational fluid dynamic (CFD) simulations were used to determine wall shear stresses. Based on these estimates, needles of 22 and 27G were selected to originate physiologically relevant wall shear stresses (Figure 7b-iv). The authors evaluated the transport of fluorescent NPs in each compartment and were able to observe, as expected, enhanced permeability effects in the vascular endothelium (a hallmark of cancer) induced by the presence of cancerous tissue. For example, vessels in the breast tumor compartments were 2.62-fold more permeable to NPs than were vessels surrounded by healthy tissues.

The recapitulation of more realistic vascularization demands the use of more sophisticated microfabrication techniques [181]. Shirure et al. designed a tumor-on-chip system that emulates physiological mass transport at the arterial end of a capillary within a tumor niche (Figure 7c-i) [56]. A key feature of this tumor-on-chip is the fabrication of a perfusable 3D microvascular network within a hydrogel compartment (Figure 7c-ii), and loading of tumor cells, cancer cell-derived tumoroids, or patient-derived tumoroids in an adjacent chamber. The authors demonstrated that nutrients and/or drugs could be effectively delivered to the tumorous tissue exclusively through the vascular network. In addition, they showed that MCF7 primary breast tumoroids could be maintained within the device for several weeks. Throughout this period, the tumoroids remained physiologically active and induced robust sprouting angiogenesis. The authors also used this platform to evaluate the effects of anticancer therapies on both patient-derived breast cancer organoids and commercial cancer cell lines seeded at high concentrations; this tumor-on-chip system recreates the dynamics of key features of tumor evolution, such as angiogenesis and cancer cell proliferation, migration, and intravasation. This work suggests vascularized tumor-on-chip systems will provide the ability to explore fairly complex precision medicine cancer scenarios in clinically relevant timeframes (≤14 days).

Agarwal et al. developed a bottom-up method to recreate vascularized 3D-tumor microenvironments [57]. First, the authors encapsulated cancer cells within hydrogel spheres to fabricate avascular tumoroids (Figure 7d-i). These microspheres were then used as building blocks to assemble a macroscale vascularized tissue by adding endothelial (and other stromal) cells in the inter-sphere spaces. Agarwal et al. found that cells could produce significantly larger cell aggregates in these 3D microenvironments than in 2D-culture systems. The cancer cells within vascularized 3D-tumors were also more resistant to DOX when compared to avascular microtumors and to 2D-cultured cancer cell monolayers (4.7- to 140-fold, respectively). High-fidelity 3D models, such as the one presented by Agarwal et al. [57], may be powerful tools that will provide insight into key processes of cancer progression that involve the role of vasculature within the tumor microenvironment.

In a recent paper, Sobrino et al. described the development, characterization, and use of in vitro vascularized microtumor environments (VMTs; Figure 7e) [58]. The authors recreated, in a geometrically simple device (Figure 7e-i), a fully perfusable and complex vasculature network and was able to demonstrate the feasibility of culturing tumor spheroids for extended time periods. Remarkably, the vasculature network was fabricated by the spontaneous self-assembly of anastomoses, which were formed by simply depositing a slurry composed of endothelial cells and extracellular matrix material (i.e., fibrinogen solution) in rhombic-shape chambers flanked by two channels and establishing a pressure differential (hydrostatic head of 5 mm H_2_O) to promote flow (see Figure 7e-i,ii). This spontaneous process occurred in a relatively short time frame. The endothelial cells rapidly proliferated and migrate into the tissue and outer channels (Figure 7e-ii). Vessel-like segments appeared within 2–3 days. Over the course of 5–7 days, the endothelial cells self-assembled into an interconnected network that spontaneously anastomosed with the outer channels. Over time, the endothelial cells also tightly lined the surface of the channels, and a fully developed network was observed by day 7. Soon after, the cells formed a tight seal and a practically leak-free vascular system was fully developed. Flow through the device switched from interstitial to intraluminal at that point (Figure 7e-iii). The authors demonstrated the feasibility of seeding and successfully culturing cancer cell aggregates (a sort of spheroid) of both breast and colorectal cancer cells in this tumor-on-chip (Figure 7e-iv). They did so by seeding a suspension of cancer cells mixed with thrombin and an extracellular mix at time zero. Thrombin contributed to the formation of tumor organoids simultaneously with the establishment and maturation of the vasculature network. Tumors and stromal cells (endothelial cells) coexisted in these microvessels networks for up to 24 days. The tumors grew vigorously in the 3D extracellular matrix, depending for survival entirely on nutrient delivery through living, fully functional, and leak-free vasculature. Remarkably, the tumors responded to standard-of-care drugs, displaying reduced growth and/or regression (Figure 7e-v). The authors demonstrated that vascular-targeting agents with different mechanisms of action could also be discriminated. For instance, anticancer compounds targeting only VEGF receptors (i.e., vandetanib and apatinib) were not effective, whereas drugs that targeted Tie-2 and PDGF and, VEGF receptors (i.e., cabozantinib and linifanib) induced a regression in the tumor vasculature (Figure 7e-vi,vii). Remarkably, HCT116-cells growing in the vascularized microfluidic tumors exhibited chemoresistance to oxaliplatin that was one order higher than that shown by cells cultured in a 2D monolayer. The VMT platform is complex in function, but simple in design, and provides a state-of-the-art model for studying vascularized solid tumors *in vitro*.

## 6. Applications: Toward Precision Medicine

**Figure 8:**
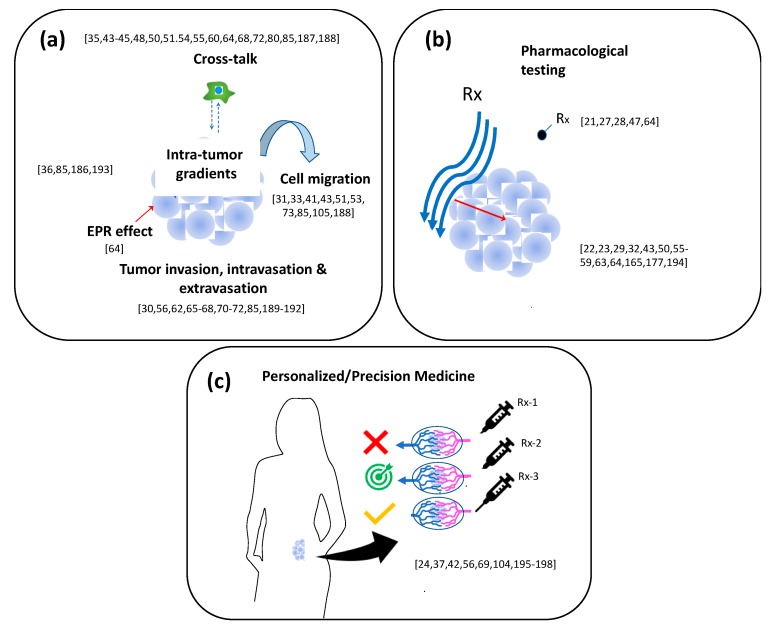
Application scenarios for tumor-on-chip systems. (**a**) Fundamental studies [30,31,33,35,36,41,43,44,45,48,50,51,53,54,55,56,60,62,64,65,66,67,68,70,71,72,73,80,85,105,186,187,188,189,190,191,192,193]; (**b**) Pharmacological screening and testing [21,22,23,27,28,29,32,43,47,50,55,56,57,58,59,63,64,165,177,194]; and (**c**) personalized/precision medicine strategies [24,37,42,56,69,104,195,196,197,198].

Tumor-on-chip systems have found niches of applications in different fronts of biomedical research and should hit the ground in clinical applications in the near future (Table 1). In previous sections, while describing the architecture of recently proposed tumor-on-chip systems, we have provided various examples of their applications in fundamental cancer research and drug testing (including NP drug delivery). In Figure 8, we have grouped recent and representative tumor-on-chip contributions into these three main spheres of applications.

As the reader may anticipate, a vast set of contributions illustrate the use of pharmaceutical anticancer compounds in tumor-on-chip systems. Many authors have found advantages related to convenience, reduced time to obtain results, easy visual access, and other comparative advantages over 2D culture or 3D static systems. In particular, anticancer nanomedicines are an important trend in cancer pharmaceutical research [185]. However, studying the dynamics of the transport of NPs from the blood to the tumor site has proven highly challenging in animal models. By contrast, valuable information has been obtained from tumor-on-chip models [21,27,28,47,64] as indicated by the following notable examples of tumor-on-chip platforms used to study NP transport and penetration into the tumor niche.

Kwak et al. studied the transport of NPs into the tumor niche, using a fairly complex tumor-on-chip, where well-defined pressure gradients could be established (Figure 4b) [28]. Their results suggested that transmembrane transport was much faster than the interstitial diffusion inside the tumor and that particle size was a key determinant of intra-tumor transport. For instance, 100 nm NPs exhibited a rapid transmembrane transport and subsequent diffusion into tumor-like tissues. As the NP size increased to 200 nm, the transmembrane transport was markedly decreased, even though the NP size was still smaller than the cut-off diameter of the membranes used in their devices. This study shows that, to ensure the effective delivery of anticancer agents to tumors, NPs need to be sufficiently smaller than the endothelium cut-off pore size [28]. Also, these results suggest that the in vivo size window for effective NP tumor uptake could be narrower than that referred in literature and mainly based on the enhanced permeability and retention (EPR) paradigm; indeed, NPs smaller than 100 nm in diameter could be more effective. Clearly, deriving these observations from in vivo experiments would have been much more challenging and costly.

Similarly, Huang et al. studied the effect of several NP properties (i.e., protein corona, size, and surface charge) on the spatiotemporal performance of NP-assisted drug delivery [21]. The authors directed polystyrene-based NPs (100 nm), displaying negative or positive surface charges, to tumor spheroids under static and flow conditions in culture medium, with or without serum proteins. Confocal laser scanning microscopy and image analysis were used to quantitate NP penetration. Results revealed that negatively charged NPs could attach more effectively to and penetrate spheroids. In addition, in experiments conducted in the presence of serum, the protein corona that formed around the NPs changed their surface properties and weakened the NP−cell affinity. This decreased the NP concentration on the spheroid surface but facilitated deeper penetration. Therefore, NP penetration in drug delivery applications may be enhanced by using NPs and less prompt to develop a protein corona and negatively charged. This work nicely illustrated an in vitro contribution to the rational design of NPs for drug-delivery application at the tumor site [21] using tumor-on-chip systems.

Contributions aimed at understanding the fundamental aspects of solid tumors (and cancer in general) are now also appearing with high frequency and are greatly meaningful. Tumor-on-chip systems have a key role in this kind of application, since some fundamental aspects of cancer can be exclusively interrogated in tumor-on-chip systems. For example, cancer is generally lethal when tumor cells become invasive and metastasis occurs, so the in vivo study of the dynamics of cancer cell invasion, from a tumor to neighboring normal tissue, is highly challenging. For example, the in vitro microfluidic model developed by Sung et al. enabled an investigation of breast cancer progression and elucidated the underlying molecular mechanisms that governed the progression of DCIS to its invasive form [33]. The use of this microscale model revealed a two-staged mechanism of invasion, where invasion progression was based first on the presence of soluble factors and then was directed by cell-cell contact.

Understanding the transition pathways of cancer is important for the development of therapeutic approaches that will inhibit its progression. Gioiela et al. developed a tumor-on-chip to study the interplay between normal and cancerous cells [35]. They used a combination of optical accessibility with a 3D tissue model in their device and were able to conduct real-time monitoring of the switch between the healthy and pathologic status *in vitro*. Another example is the initiation and progression of the ALL as it occurs in the bone marrow. The bone marrow microenvironment is complex in terms of the cellular and ECM constituents. The bone marrow cell population is very heterogeneous, consisting of hematopoietic cells and stromal cells, including fibroblasts, adipocytes, macrophages, and osteoblasts. The in vitro recreation of the bone marrow microenvironment is important for understanding cancer progression, relapse, and chemotherapy effectiveness. Bruce et al. developed a 3D microfluidic cell culture platform where some characteristics of bone marrow, including tissue density and blood rate flow, could be recapitulated [49]. Tang et al. [64] studied the EPR effect [199] in a tumor-on-chip system and showed that endothelial cells co-cultured with metastatic MDA-MB-231 cancer cells formed leakier vascular channels than those co-cultured with non-metastatic MCF-7 cancer cells.

The application of tumor-on-chip systems in precision or personalized medicine deserves particular mention. Some recent papers have described the maintenance of portions of real tumorous tissue from biopsies in microfluidic systems [104,197,198,200]. For instance, Bower et al. have described an experiment in which they were able to sustain the viability of head and neck cancerous tissue for 48 h in a simple microfluidic chamber [197]. The authors promoted a laminar flow regime (Re~10^−3^) around small tumor samples (5 to 10 mg) and evaluated the morphology, viability, and proliferation capability of these samples. They found no significant differences prior to and at 48 h post-culture.

Astolfi et al. cultured microdissected tissues (MDTs), at dimensions below diameters of ~420 μm, on a simple microfluidic platform [198]. The MDTs were cultured and kept alive on the chip in a low-shear stress environment for extended time periods (over one week). The authors conducted perfusion experiments using tissues (including one sample from a patient with benign prostatic hyperplasia and some mouse xenografts derived from ovarian and prostate cancer patients). The MDTs were analyzed by flow cytometry and confocal microscopy over an incubation period of eight days. In addition, the authors conducted a proof-of-principle anticancer drug testing experiment under continuous perfusion of human tissue from a cancer patient.

Sylvester et al. cultured head and neck squamous cell carcinoma (HNSCC) tumor biopsies in a microfluidic device with the aim of generating clinically relevant information [104]. The authors conducted experiments in which fresh or cryogenically frozen primary HNSCC or metastatic lymph node samples were exposed to anticancer drugs (i.e., docetaxel, 5-fluorouracil, or cisplatin). They found that proliferation and cell death were statistically similar in the frozen tissue and fresh samples. In addition, all three drugs caused cell death in a dose-dependent manner. Drug combinations exhibited the highest cytotoxicity, in agreement with published clinical data.

The development of cancer therapies based on precision medicine can only be effectively accomplished if individual tumors can be rapidly tested for therapeutic sensitivity in clinically relevant timeframes (i.e., ≤ 14 days). Some studies have demonstrated that this goal can be achieved, and that precision medicine based on the use of tumor-on-chip systems is not simply a promise but a reachable outcome of the integration of microfluidics and tissue engineering. For example, Shirure et al. demonstrated the feasibility of using patient-derived tumor organoids in a tumor-on-chip device to conduct personalized medicine studies [56].

## 7. Challenges and Perspectives

Cancer research has greatly benefited from the use of 3D culture systems in the last decade. Nevertheless, better 3D tumor models are still required to obtain a more faithful recapitulation of the high complexity of human tumors [103]. Among 3D culture systems, the so-called tumor-on-chip devices are at the forefront in terms of achieving a proper recapitulation of human tumor microenvironments.

The possibility of combining tissue engineering tools and microfluidics into tumor-on-chip systems enables finer and deeper cancer research while minimizing time, cost, and ethical concerns when compared to research done using animal models. Moreover, tumor-on-chip platforms open the possibility of using human cells and human microtissues, which is highly appealing and relevant to human medicine. Arguably, research conducted in human tumor-on-chips should be closer to clinical translation than are preclinical studies done in conventionally cancer-research models based on the use of murine cells or animal models. The use of tumor-on-chip systems provides a novel and powerful tool to gain insight into the physiology of human tumors and to study the effects of pharmaceutical compounds before resorting to animal models.

Tumor-on-chip systems put humankind at the threshold of designing personalized, high-precision cancer medicines. In principle, an actual biopsy taken from a patient could be expanded into multiple spheroids (tumoroids) that could be cultured in tumor-on-chip systems for cost-effective screening for therapies particularly efficacious for that patient. However, before we get to that point, many terrain issues need to be resolved. For example, the fabrication of microfluidic devices is still a task that requires skill and experience [12,201]. In the years to come, the incorporation of high-resolution 3D printing, better biomaterials, and more sophisticated tissue engineering techniques for develop vasculature will greatly contribute to more rapid advancements in the development of more physiologically relevant tumor-on-chip systems. The advent of high-resolution 3D-printing technologies will enable the easier fabrication of more complex and functional organ-on-chip devices [202,203]. In turn, this will “democratize” the development of organ-on-chip systems. Some recent examples illustrate this in the very context of tumor-on-chip applications [204].

Among all emerging biofabrication techniques, we would argue that bioprinting [205] holds the highest promise to make a decisive contribution to improve the engineering of tumors for cancer research [206]. For example, today, several biofabrication steps are needed to create a tumoroid surrounded by healthy tissue. An even greater challenge is to develop vascularization around (or inside) the tumor. Here, novel biofabrication strategies will play a decisive role [207]. In particular, 3D bioprinting will be also a valuable asset [208,209]. Although the resolution and speed of this generation of commercial bioprinters is still limited, several multi-material bioprinting approaches [210,211,212] will certainly simplify the fabrication of complex tumorous tissues [206,213]. Recent papers demonstrate the extraordinary potential of bioprinting for better engineering of the tumor niche [93,209,214,215,216,217,218,219] and better recapitulation of cancer progression or anticancer drug effectiveness. For example, Wang et al. bioprinted constructs that contained breast cancer cells within a central core surrounded by adipose-derived mesenchymal stem/stromal cells (ADMSC) [93]. Trujillo-de Santiago et al. used chaotic bioprinting, a microfabrication technique based on the use of chaotic flows, to fabricate a construct containing a high amount of interface between cancerous and normal cells [210].

CFD is another tool that, although not yet frequently used, promises to be of great help in addressing the different aspects of the design of tumor-on-chip systems. Several recent contributions demonstrate the use of CFD simulations to study the transport of materials near a tumor in tumor-on-chip systems [26,56] or in vivo [220]. Several other papers have illustrated the use of CFD to improve the hydraulic design of lab-on-chip systems [221] and organ-on-chip systems [69,222], including tumor-on-chip devices.

While the dynamics of tumor growth is local, cancer is a systemic disease. Many more patients die today from metastatic cancer than they do from their original tumors [17]. Human-on-chips systems that recapitulate not only the tumor niche but also one or more possible target metastasis organs connected throughout a vascular/fluidic network—will be a highly valuable tool for proper modeling of metastasis [99,223,224]. Pioneering examples of human-on-chip systems to model tumor metastasis have been recently published [67,192,225].

The complete recapitulation of the true nature and evolution of a malignant tumor in vitro is challenging, and a high-fidelity 3D emulation of the tumor microenvironment remains an aspiration for fundamental studies in cancer research, for anticancer drug discovery and screening [57], and for the development of precision medicine strategies. Tumor-on-chip systems will be protagonists in cancer research in the years to come, progressively more assisted by novel microfabrication technologies, more precise instrumentation, single-cell omics, and systems biology approaches. However, this sophistication is not always cost-effective—or even required—for some specific applications. In the years to come, we will continue to see simple and elegant embodiments of the concept of tumor-on-chip and the tremendous potential of tumor-on-chip systems to provide insights into cancer biology.

## Figures and Tables

**Table 1:** Summary of a selection of recently published research papers (2013–2019) on tumor-on-chip systems.

Description/Flow Conditions	Cancer Type/Cell Line	Materials (Microfluidic Device)	Biomaterial (ECM)	Application	Reference
Microfluidic channel with multiple micro traps for individual spheroids (Figure 2). Nutrients diffuse from the main channel to trapped spheroids. Flow rate (main channel): 100 µL/h	malignant pleural mesothelioma (MPM) cells	PDMS	No extracellular matrix (ECM) was used	To compare the therapeutic efficiency of cisplatin, a drug commonly prescribed for MPM patients, and compare chemoresistance of cancer spheroids exposed to cisplatin in static (*ex-device)* versus dynamic environments.	[20]; Figure 3a
Triple layer microfluidic conduit in which spheroids are captured in semicircular traps for growth/maturation.	Human Hepatic cancer; HepG2	PDMS (top layer); glass (middle layer); PVC (bottom layer)	Non-embedded spheroids	To study nanoparticle penetration into tumor spheroids: The effects of protein corona, protein size, and charge were analyzed.	[21]; Figure 3b
Simple, bi-layered, and pump-independent microfluidic system devised for continuous formation and perfusion of cancer spheroids contained within rectangular cavities located on the floor of the microfluidic circuit. Flow is driven simply by a pressure head induced by a difference in the height of the column of liquid (culture medium) between the inlet and the outlet reservoir.	Human hepatic cancer; (HepG2)	PDMS	Non-embedded spheroids	To study the process of formation of hepatic cancer spheroids under continuous perfusion. Spheroids are formed within cavities in the bottom floor of the device, and cavities of two different sizes are tested. To test the anti-cancer effects of three compounds (tirapazamine, cisplatin, and resveratrol) on hepatic cancer spheroids.	[22]; Figure 3c
Microfluidic chamber (Figure 3) composed of a central compartment and two side channels: the central channel was filled with cancer spheroids embedded in gelatin (cross-linked using glutaraldehyde); the two side channels were used for continuous feeding of liquid streams at 30 µL/h (0.5–30 µL/min). (The linear speed was ~278 µm/s). Breast cancer spheroids, prepared *ex-device* by an overflow method, were embedded in the gelatin-filled central compartment.	Breast Cancer; MCF7 cells	PDMS	Gelatin cross-linked using glutaraldehyde	To evaluate therapeutic efficiency of doxorubicin (DOX), an anthracycline antibiotic that intercalates DNA, in multi-cellular tumor spheroids (MTS) fabricated *ex-device.*	[29]
Microfluidic system composed of an inlet channel connected to a visualization chamber, where a tumor spheroid is physically trapped by slight compression against a glass coverslip.	Human breast cancer; MDA-MB-435 cells	PDMS and glass coverslip	Non-embedded spheroids	To study the transport, penetration, and accumulation of nanoparticles in cancer spheroids in real time (under a microscope)	[27]; Figure 4a
Microfluidic device formed by two stacked layers of microchannels with a porous membrane sandwiched between the layers. The top layer has a channel simulating the capillary of the tumor vasculature. The endothelium of the capillary is mimicked by culturing MVECs on the porous membrane. The bottom layer has three channels, which are partitioned with periodically placed posts. The center channel simulates the tumor surroundings where spheroids dispersed in collagen are placed, and the two side channels simulate the lymphatics. In the tumor channel, cancer cells grow within a 3D collagen matrix, while the interstitial fluid flows through the matrix and creates an elevated interstitial fluid pressure. Nanoparticles are transported through this 3D tissue structure and reach the cancer cells.	Breast Cancer; (MCF7 cells) and endothelial cells (MVECs)	PDMS (layers); polycarbonate membrane	Collagen I and Matrigel (for the membrane coating)	To simulate the complex transport of nanoparticles around a tumor spheroid in a TOC system, where well-defined pressure gradients can be established. The authors studied the effect of size, concentration, and dynamic conditions in targeted delivery of anti-cancer compounds encapsulated in nanoparticles	[28]; Figure 4b
Y-shape device with two microchannel lines that enable the co-culture (sharing an interface) of mammary epithelial cells (MCF-DCIS) and non-cancerous human mammary fibroblasts (HMFs) (Figure 5). Sample loading and fluid changes are performed using a surface-tension driven pump.	Breast cancer; Mammary epithelial ductal carcinoma in situ cells (MCF-DCIS)	PDMS	Hydrogel mix: 1:1 matrigel and collagen I (0.8 mg/mL)	To evaluate the progression of breast cancer cells from ductal carcinoma in situ (DCIS) to invasive ductal carcinoma (IDC)	[33]; Figure 5a
Microfluidic chip composed of two compartments for micro-tissue (3D-µTP) accommodation: the inner chamber is for the tumor and the outer one for the stromal compartment. The chambers are separated by an interface that allows physical contact. The two chambers have a dedicated channel for cell culture loading, while the other two side channels allowed the flow of culture medium at a nominal flow rate of 3.0 µL/min.	Breast cancer; Normal mammary Fibroblasts (NF) and Cancer Associated Fibroblasts (CAF), Human breast adenocarcinoma cells (MCF7)	PDMS	ECM produced by micro-tissues	To replicate in vitro the stromal activation that occurs during tumor epithelial invasion	[35]; Figure 5b
Microfluidic device to study a tumor microenvironment in real time. This tumor-on-chip consisted of a central microchamber flanked by two lateral microchannels. Tumor cells were embedded within a collagen matrix in the central microchamber (Figure 5d-i,ii), while the lateral microchannels were used to perfuse medium, oxygen, and anticancer drugs. The configuration of the central chamber enables the spontaneous generation of normoxic, hypoxic, and necrotic regions within the device.	Two cancer cell lines in independent experiments—glioblastoma U-251 MG cells and HCT-116 cells from colon carcinoma	polystyrene	Collagen	To establish clear oxygen gradients and hypoxic conditions in a microfluidic device and to study the effect of anticancer drugs (DOX for colon carcinoma and TMZ for glioblastoma) in cell populations under controlled oxygen gradients, without resorting to the use of spheroids.	[36]; Figure 5d
Four-unit microfluidic chip. Each unit consists of three cell-loading channels (to fill with cells in collagen) and four medium channels (Figure 6-i–iv). Channel width was 1000 μm and channel depth was approximately 190 μm; material/gas exchange was accommodated between the channels.	Normal colon fibroblasts (CCD-18Co) and human colorectal cancer cells (HT-29 cells)	PDMS	Collagen Type 1	To study the crosstalk and mutual activation between fibroblasts and cancer cells. To evaluate the effect of different doses of DOX in monoculture and co-culture conditions within this TOC system.	[51]; Figure 6b
Commercial device: ‘3-D cell culture chip’ (DAX-1) from AIM BIOTECH: https://www.aimbiotech.com/. Each single layer slide format chip (75 mm × 25 mm), consisting of 3 microfluidic chambers, each with a central gel channel (width 1.3 mm) flanked by two medium channels (width 0.5 mm). The height of the microfluidic chambers is 0.25 mm. (Figure 3a–c).	Patient-derived spheroids from real tumor samples: Samples contained cancerous cells, stromal cells and immune cells.	cyclic olefin polymer (COP)	Collagen Type 1 (rat tail)	Patient-derived spheroids (PDOSTS) were cultured in contact with immune system cells to recreate the tumor immune microenvironment and screen the response of tumors to immunotherapy (therapies based on antibodies)	[48]; Figure 6c
3D microfluidic device that allows co-culture. The device comprises four perfusion microchannels with only one inlet and one outlet. The cells are loaded and culture medium is fed using two ports (“cell in” and “media in”) placed at the inlet side of the device. The velocity rate within the microchannels was 0.27 ± 0.18 μm/s.	Acute lymphoblastic leukemia (SUP-B15 cells), and bone marrow mesenchymal stem cells (BMSC).	PDMS	Collagen Type 1	To elucidate cell–cell and cell–matrix interactions on leukemia progression and to test therapeutic agents in a co-culture.	[49]; Figure 6d
Dual compartment human-on-chip system composed of a liver compartment (healthy or tumorous), and a breast cancer compartment. Each compartment (volume ~0.5 cm^3^) contains a microtissue formed by a mixture of cells (liver or breast cancer cells) in collagen. Compartments are connected by a vascular (endothelialized) channel that crosses through each microtissue (Figure 7b-i–ii).	Liver or breast cancer; Human breast cancer cells (MDA-MB-231); healthy liver cells (THLE-3); carcinoma liver cells (C3Asub28); and telomerase immortalized microvascular endothelial (TIME) cells.	PDMS and glass cover	Collagen Type I: at 7 mg/mL (for cancerous tissues) and at 4 mg/mL (for healthy tissues)	To recapitulate an interactive liver–tumor tissue microenvironment on a chip for the investigation of nanoparticle transport and toxicity.	[55]; Figure 7b
Microfluidic platform consisting of a series of three rhombic tissue chambers (Figure 7e-i–iii). These are connected to two adjacent channels by two capillary burst valves that retain a mixture of cells and ECM inside the chambers. At the two ends of the tissue chambers are two gel-loading ports for introduction of introduced the cell–ECM suspension. Four media reservoirs are attached to the inlets and outlets of the microfluidic channels.	Three colorectal cancer cell lines (HCT116, SW620, and SW480); two breast cancer lines (MCF-7 and MDA-MB-231), and a melanoma cell line (MNT-1). Endothelial cells.	PDMS and glass cover	A mix of fibrinogen in PBS with Ca^2+^/Mg^2+^ to a final concentration of 10 mg/mL; and thrombin (30 U/mL) and laminin (1 mg/mL)	To develop a vascularized TOC and recreate a vascularized environment relevant to the progression of cancerous tissue and the testing of anti-cancer agents. To demonstrate that vascular-targeting agents with different mechanisms of action (i.e., VEGF blockers, vascularization inhibitors, etc.) can be distinguished in this TOC. For example, to show in vitro that drugs targeting only VEGFRs (i.e., apatinib and vandetanib) are not effective, whereas drugs that target VEGFRs, PDGFR, and Tie2 (i.e., linifanib and cabozantinib) do regress the vasculature.	[58]; Figure 7e
